# Self-assembled liquid crystal architectures for soft matter photonics

**DOI:** 10.1038/s41377-022-00930-5

**Published:** 2022-09-13

**Authors:** Ling-Ling Ma, Chao-Yi Li, Jin-Tao Pan, Yue-E. Ji, Chang Jiang, Ren Zheng, Ze-Yu Wang, Yu Wang, Bing-Xiang Li, Yan-Qing Lu

**Affiliations:** 1 National Laboratory of Solid State Microstructures, Key Laboratory of Intelligent Optical Sensing and Manipulation, College of Engineering and Applied Sciences, and Collaborative Innovation Center of Advanced Microstructures, Nanjing University, Nanjing, 210023 China; 2 College of Electronic and Optical Engineering & College of Flexible Electronics (Future Technology), Nanjing University of Posts and Telecommunications, Nanjing, 210023 China

**Keywords:** Liquid crystals, Displays

## Abstract

Self-assembled architectures of soft matter have fascinated scientists for centuries due to their unique physical properties originated from controllable orientational and/or positional orders, and diverse optic and photonic applications. If one could know how to design, fabricate, and manipulate these optical microstructures in soft matter systems, such as liquid crystals (LCs), that would open new opportunities in both scientific research and practical applications, such as the interaction between light and soft matter, the intrinsic assembly of the topological patterns, and the multidimensional control of the light (polarization, phase, spatial distribution, propagation direction). Here, we summarize recent progresses in self-assembled optical architectures in typical thermotropic LCs and bio-based lyotropic LCs. After briefly introducing the basic definitions and properties of the materials, we present the manipulation schemes of various LC microstructures, especially the topological and topographic configurations. This work further illustrates external-stimuli-enabled dynamic controllability of self-assembled optical structures of these soft materials, and demonstrates several emerging applications. Lastly, we discuss the challenges and opportunities of these materials towards soft matter photonics, and envision future perspectives in this field.

## Abstract (web summary)

This paper reviews recent advances in self-assembled liquid crystalline architectures in terms of their fabrications, manipulations, as well as emerging applications.

## Graphical abstract

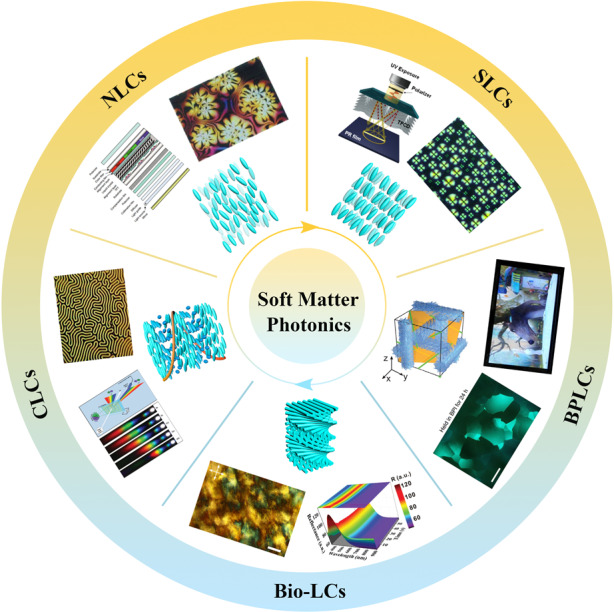

## Introduction

“Soft matter” is firstly proposed by Pierre-Gilles de Gennes in his Nobel acceptance speech in 1991, which describes materials between aqueous substances and ideal solids, such as colloids, foams, liquid crystals (LCs), gels, polymers, and active matter^1,2^. Soft matter materials lay the advantageous foundations of living systems in nature due to their spontaneous self-assembly of functional organizations and the superior ability to sense, function, and response to various environmental stimuli^3–6^. The weak interaction among soft building blocks triggers a fragile balance between entropic and enthalpic contributions to the free energy^7–9^, which facilitates the self-assembly of multiple length-scale microstructures^8,10,11^ with phenomena closely related to both the inherent characteristics of nanomaterials and the structural engineering of building blocks throughout a spatial region^3,12,13^. For instance, chameleon exhibits a rapid and reverse shift of color patterns when interacting with the outside by actively structuring non-close-packed guanine nanocrystals within the skin^14–16^. Till now, remarkable soft materials with a wide variety of complex configurations^17^, colorful patterns^18^, metastable states^11^, and macroscopic softness^19,20^ have provided valuable inspirations for addressing modern challenges in wide ranges of areas^21–23^, especially in advanced optical and photonic technologies^24^, driving the development of soft matter photonics.

LC represents one of the most attractive soft matter systems^25–31^. In living organisms, biocomponents including proteins, deoxyribonucleic acids (DNAs), polysaccharides, and lipids are kept in LC states through well-defined self-assembly processes, which play important roles in plentiful life activities, including metabolism, information delivery, and interoceptive awareness^32,33^. As the name indicates, LCs possess both the fluidic property of conventional liquids and the ordering nature of crystals^1,6,34–36^. They can achieve intriguing and programmable hierarchical superstructures with a high sensitivity to external stimuli, such as electric field^37,38^, light exposure^39,40^, magnetic field^41^, mechanical action^42^, and interface conditions^43,44^. The anisotropic molecular structures, combined with the long-range orientational order and adaptive stimulus-responsiveness, endow LCs with desirable birefringent optical performance^45,46^. This feature makes LCs an unfailing paradigm for display industries^45,47^, with the annual value of production reaching hundreds of billions of dollars. From this point of view, it is believed that, as a crucial material to life itself and displays manufacture, LCs show great potentials to promote the thriving topic of soft matter optics^48–52^. On the other hand, LCs derived from biomass (such as cellulose, DNA, tobacco mosaic virus, chiral polypeptide LC solvent, and silk) are appealing candidates for developing soft and sustainable optical platforms^53,54^. Naturally derived bio-based LCs provide new opportunities towards the replacement of existing non-renewable optical platforms with renewable, biocompatible, and biodegradable systems that match the high performance of their synthetic counterparts, while minimizing waste, environmental degradation, and energy-intensive input^55^. Additionally, the features of hierarchical and tailorable structures, stimuli-responsiveness, functionalized capabilities, and facilitation of formation of different material formats make them ideally suitable for soft and smart photonic materials.

**Fig. 1:**
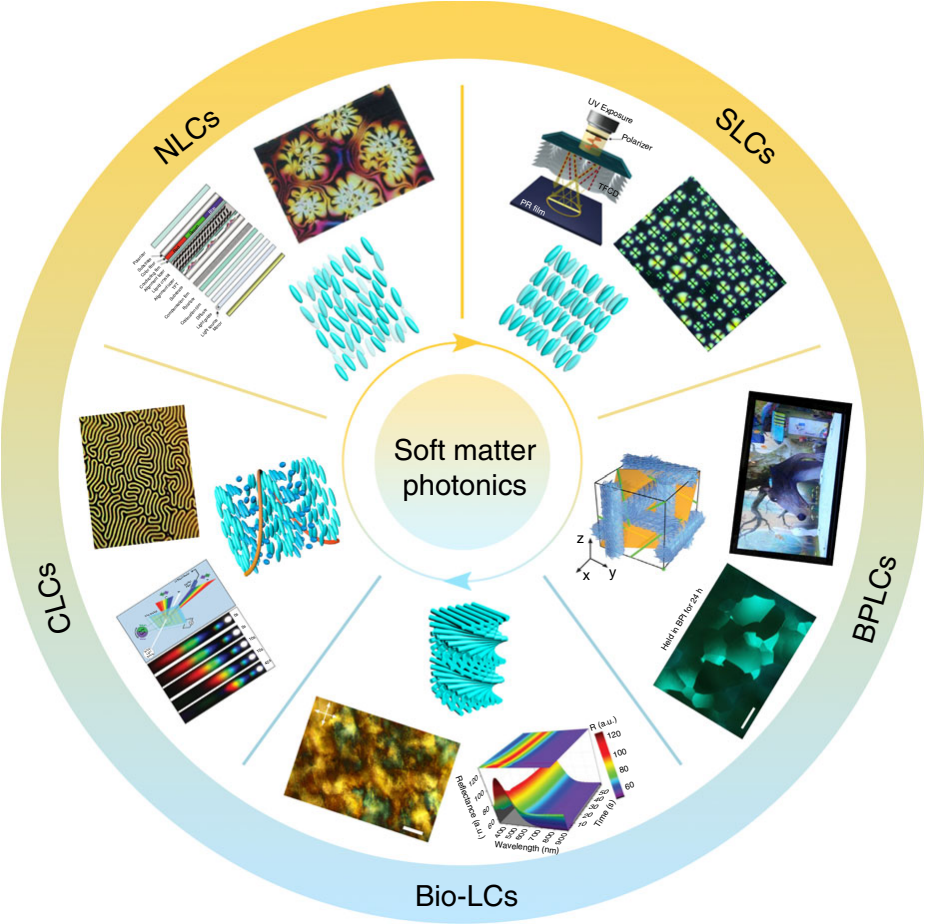
Soft matter photonics. Schematic structures, representative textures, and promising applications of soft matters including nematic phase LCs^6,7^ (Reproduced from refs. ^6,7^, with permissions from John Wiley & Sons and AAAS), smectic phase LCs^96^ (Reproduced from ref. ^96^, with permission from Wiley-VCH), cholesteric phase LCs^296,297^ (Reproduced from refs. ^296,297^, with permissions from Wiley-VCH), blue phase LCs^144,298,299^ (Reproduced from refs. ^298,299^, with permissions from Wiley-VCH. Reproduced from ref. ^144^, with permission from Springer Nature: Nature Communications), and bio-based LCs^183,203^ (Reproduced from refs. ^183,203^, with permissions from Springer Nature: Nature Communications and Wiley-VCH)

Over the past years, optical systems based on LCs (typical thermotropic and bio-based lyotropic LCs) have experienced a booming development, promoting the emergence of new phenomena, functions, and applications. In this article, we present recent advances in the fabrications, manipulations, and applications of self-assembled optical LC architectures, Fig. 1. We first summarize the basic properties of typical thermotropic LCs and bio-based lyotropic LCs, i.e., nematic phase LCs, smectic phase LCs, cholesteric phase LCs, blue phase LCs, and celluloses. Next, we analyze the manipulation schemes of LC architectures, especially the topological defects and topographic configurations, with an emphasis on the dynamic control of these self-assembled optical structures. Subsequently, we describe several burgeoning optical and photonic applications, such as smart displays, optical imaging, and light field modulation devices. In the last part, we discuss the challenges and opportunities of these systems towards soft matter photonics, and provide visions for the future perspectives in this field.

## Typical thermotropic liquid crystal architectures

Microstructures bridge the inherent properties of nanomaterial and the important functionalities of devices. To develop ideal LC-based devices, the priority is to tailor long-range ordered LC microstructures. Many efforts have been devoted into this field to creating on-demand LC superstructures with wide tunability. In this section, we focus on different LC phases, including nematic, smectic, cholesteric, and blue phase LCs, and describe the judicious control and dynamic modulation of LC microstructures, especially topological defects. The central idea relies on the stimulus-controlled self-organization of LC building blocks.

### Patterned structures in nematic phase LCs

Nematic LCs (NLCs), as the simplest state among various LC phases, are famous for the prevalent applications in displays^45,56,57^, due to their self-assembled long-range orientational ordering and fast response capability under electric fields^6,58–61^. Distinct from ordinary liquids, NLCs are optically uniaxial materials with anisotropic structural and physical properties (e.g., birefringence and dielectric anisotropy)^1,6^, Fig. 1, and possess the ability to form intriguing microstructures sensitive to external conditions^26,62,63^. All these unique features provide potential opportunities for NLCs in areas beyond displays, especially in optics and photonics^64^.

**Fig. 2:**
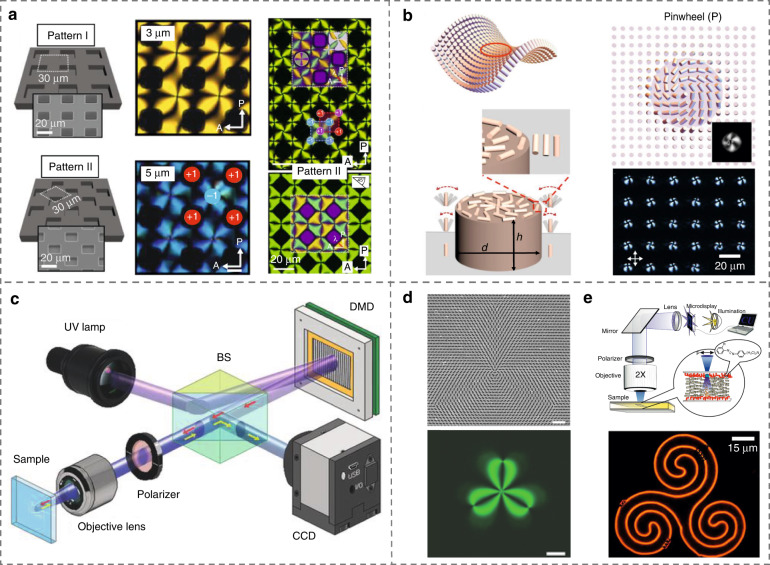
Patterned nematic structures by 3D topographic and photoaligned substrates. **a** Topological defect arrays controlled by patterned air pillars^65^. Adapted from ref. ^65^, with permission from AAAS. **b** Topographically and chemically patterned saddle-splay microstructures^66^. Reproduced from ref. ^66^, with permission from Springer Nature: Nature Communications. **c**-**e** Photopatterning of NLCs enabled by DMD-based micro-lithography setup^67^, plasmonic metamask^72^, and functional azobenzene-containing surface monolayers^73^, respectively. Reproduced from ref. ^67^, with permission from Wiley-VCH. Reproduced from ref. ^72^, with permission from Wiley-VCH^73^. Reproduced from ref. ^73^, with permission from National Academy of Sciences

The creation of elaborate patterns in NLCs is challenging. To achieve this, Kim et al.^65^ employed a Si substrate with a periodic lattice of square and round air pockets to generate ordered topological defect arrays, Fig. 2a. By this means, stable pinwheel-like birefringent textures with vibrant birefringent colors are induced and their positions were well imposed by the three-dimensional (3D) surface topography of substrates. Xia et al.^66^ reported a self-organized subtle saddle-splay arrangement of NLCs by introducing 3D topographic substrates with chemical patterns, Fig. 2b. The elastic constant *K*_24_ was identified, which enters the Frank-Oseen free energy density for both chiral and achiral materials, and carries important insights into the nature of soft materials^2^.

To add more degrees of freedom of NLC arrangement, photoalignment is further proposed based on photoresponsive alignment materials, the sulphonic azo-dye SD1 for instance^67,68^, which can reorient their absorption oscillators perpendicular to the polarization of ultraviolet (UV) light due to the isomerization of azo groups and dichroic absorption of chromophores. A digital-micromirror-device (DMD) based photopatterning system was further developed to generate arbitrary photoalignments for programmable multi-domain LC microstructures^67,69–71^, Fig. 2c. By utilizing a projection system with engineered plasmonic meta-masks, Guo et al.^72^ achieved the high-throughput and high-resolution complex LC orientations, e.g. a flower pattern with a cluster of four topological defects as shown in Fig. 2d. Moreover, Martineza et al.^73^ realized dynamic high-resolution patterning and re-patterning by adopting an azobenzene-containing surface monolayer, Fig. 2e.

**Fig. 3:**
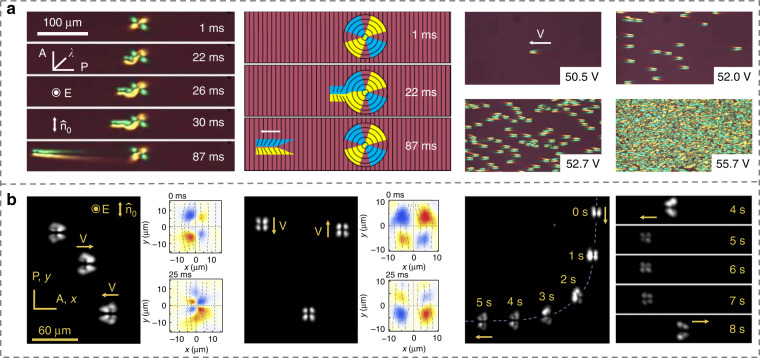
Controls of dynamic architectures in NLCs. **a** Electrical generation of 3D NLC director bullets^78^. Adapted from ref. ^78^, with permission from Springer Nature: Nature Communications. **b** Dynamic steering of LC solitons^79^. Adapted from ref. ^79^, with permission from Springer Nature: Nature Communications

Desired dynamic microstructures can also be achieved by taking advantages of the stimuli responsiveness of NLCs^74–77^. For instance, Li et al.^78^ electrically produced a series of intriguing microstructures forming 3D particle-like propagating solitary waves, Fig. 3a, which are also called director bullets, in analogy with light bullets (3D optical solitons). These bullets can survive collisions with restored shapes and velocities. Director bullet represents the distortion of LC directors, which periodically oscillates following the same frequency of electric fields. The underlying mechanism is the flexoelectric effect where the director distortion produces flexoelectric polarization. The coupling of the polarization and the electric field generates a Coulomb force which balances the viscosity dragging force. By changing the amplitude and/or frequency of the electric field, these solitons with tunable steering directions were reported^79^, Fig. 3b, enabling promising applications such as information delivery and micro-cargo manipulations^75^.

### Topological defect structures in smectic phase LCs

Smectic phase is another important mesophase among thermotropic LCs^80,81^. Distinguished from NLCs, lamellar smectic LCs (SLCs) present both the long-range orientational order and positional order^82^, i.e., the long axes of molecules perpendicular or slightly tilted to the layer plane, Fig. 1. There are abundant topological superstructures with distinct morphologies discovered in SLCs under different external conditions^83–85^. One of the most eminent topological microstructures is called the focal conic domain (FCD)^2,82,86–90^, in which smectic layers are curved with a constant interlamellar spacing typically in molecular scales and wrapped around a pair of conjugate defect lines (an ellipse and one of the hyperbolae)^91–93^.

**Fig. 4:**
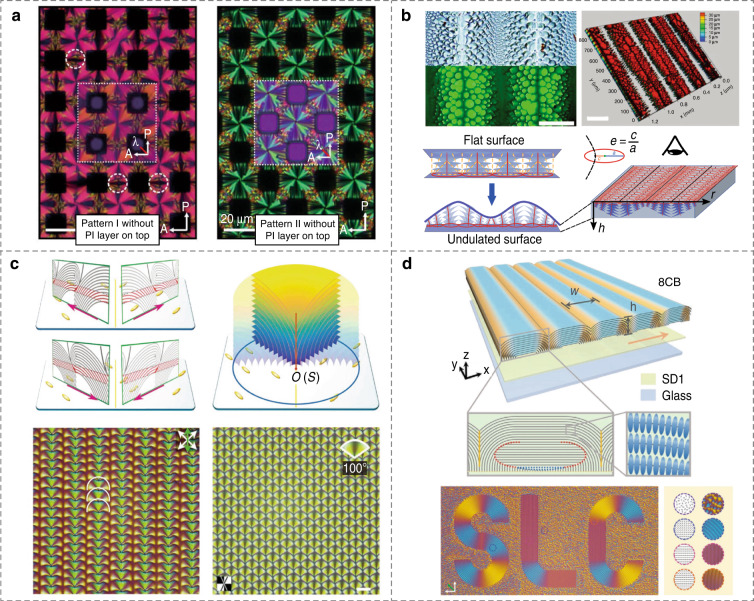
Tailoring of smectic topological defects by geometric confinement and photoalignment. **a** Topographically patterned kaleidoscopic textures^65^. Reproduced from ref. ^65^, with permission from AAAS. **b** Hierarchical assembly of FCDs by undulated surfaces^100^. Reproduced from ref. ^100^, with permission from Royal Society of Chemistry. **c** Photopatterned topological FCDs^95^. Reproduced from ref. ^95^, with permission from Wiley-VCH. **d** Photopatterned oily streaks^101^. Reproduced from ref. ^101^, with permission from Wiley-VCH

Much progress has been made to manipulate the geometric parameters of FCD arrays, including the size, shape, eccentricity, and lattice symmetry^94,95^, which brings diversified optical functionalities, e.g., microlensing^96–98^, vortex beam generating^99^, and optically selective masks^96^. Lavrentovich et al.^86^ studied the magnetic and surface anchoring effects on the nucleation and growth of smectic FCDs in 1994. By utilizing 3D topographic substrates with periodic pillars and undulated surfaces, absorbing kaleidoscopic textures and hierarchical topological FCDs were self-assembled^65,100^, Fig. 4a, b. For simultaneously dictating the geometry and clustering characteristics of FCDs, Ma et al.^95^ demonstrated the “smectic layer origami”, where 3D construction of anisotropic defects is realized by two-dimensional (2D) preprogrammable photopatterning. These microstructures break the rotational symmetry while maintaining the radially gradient director field of LCs, enabling a metasurface-like polarization-selective diffraction, Fig. 4c. In addition, defect walls of smectic oily streaks were arbitrary manipulated and flexibly modulated by combining the photoalignment and electric field^101^, Fig. 4d, making it possible for more creative functional microstructures in SLCs.

**Fig. 5:**
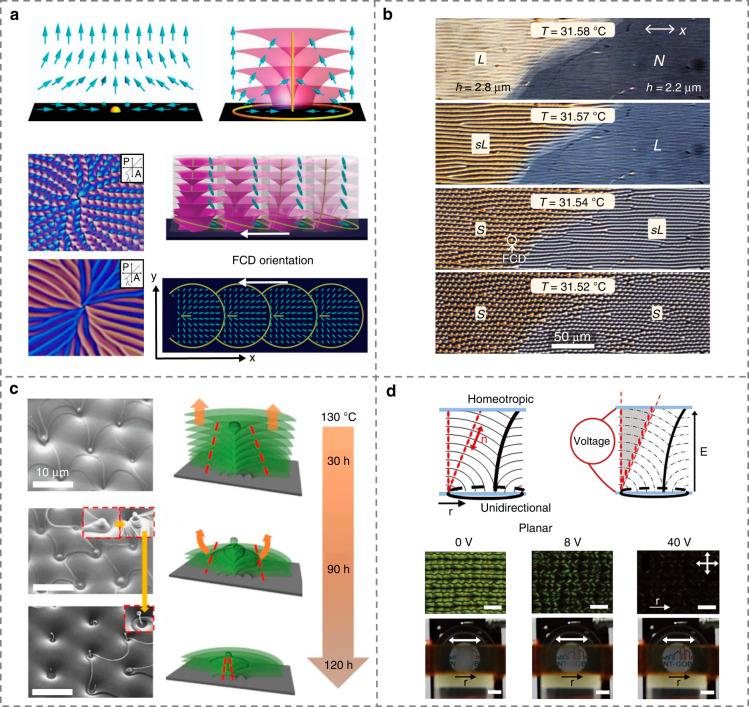
Manipulations of SLC topological defects by thermal and electric fields. **a** and **b** Dynamic defect morphogenesis during nematic-smectic phase transition^102,103^. Reproduced from ref. ^102^, with permission from Springer Nature: Nature Communications. Reproduced from ref. ^103^, with permission from National Academy of Sciences. **c** Sintering-induced Udumbara flower-like microstructures^105^. Reproduced from ref. ^105^, with permission from Springer Nature: Nature Communications. **d** Electrically reversible tuning of smectic FCDs^107^. Reproduced from ref. ^107^, with permission from Wiley-VCH

Temperature is a crucial parameter in the fine control of hierarchical smectic topological defects. Gim et al.^102^ reported the dynamic defect morphogenesis during the in situ nematic-smectic phase transition by accurately controlling the temperature of LC droplets on the water, Fig. 5a. It was found that the director field geometry of nematics would strongly affect the geometry of subsequent smectic topological defects during the phase transition, which is rationalized by illustrating the similarity of ordering between nematics and smectics. Zappone et al.^103^ examined the smectic pattern conversion from one-dimensional (1D) streaks to 2D fan-shaped FCD arrays when cooling from nematics to smectics, Fig. 5b. The long-standing prediction of an “intermediate” LC state based on an analogy with superconductors was further confirmed, which was first pointed by de Gennes in 1972^104^. By leveraging the sublimation and condensation of SLCs, Kim et al.^105^ reported an intriguing morphology transformation of FCDs with nontrivial LC curvatures, Fig. 5c. Defect structures with significant positive Gaussian curvature of smectic layers were obtained, implying a lucrative thermal control of both the mean and Gaussian curvature of SLCs. The study of SLC sublimation was recently explicated by Vitral et al.^106^ on smectic-isotropic interfaces. In addition, Boniello et al.^107^ proposed electrically reversible and dynamically tunable defect patterns in polymer-stabilized SLCs, which overcomes the long-term intractable challenge, i.e., the highly ordered SLC is essentially irreversible under electric fields, Fig. 5d. Hence, a reversible switching between different microstructures and optical states of SLCs could be achieved. The flexible manipulation and tailoring of abundant topological defects in SLCs may blaze a trail in the fields of topology, self-assembly, structural patterning, and so on.

### 1D helical structures in cholesteric phase LCs

Chirality is pervasive in nature, ranging from neutrinos to nucleic acid^108^, seashells, and galaxies^109^, which brings nontrivial phenomena and attracts scientists across various domains to explore artificial chiral nanoarchitectonics^34,110–112^. Cholesteric LCs (CLCs), also called chiral nematic LCs, exist ubiquitously in organisms^36^. They can self-organize into various elegant helical structures, Fig. 1, including fascinating fingerprints, planar textures, distinctive microshells as well as microgrid chiral structures. The helical pitch *p*, defined as the distance of a full turn rotation of anisotropic LC molecules^113^, is a decisive parameter for the helical microstructures, which can be flexibly tuned by different external stimuli such as temperature^114^, light^115^, electric field^116^, magnetic field^117^, mechanical stress^118^, and chemical conditions^119^. Intriguingly, when the periodicity of the helical structure of CLC is comparable with visible wavelengths, Bragg reflection occurs with a strong wavelength/polarization selectivity. The central reflection wavelength is

1 $$\lambda _c = \bar n \cdot p$$

where $\bar n$ is the average refractive index of LCs^1,120^. Thus, CLCs are also known as soft photonic crystals, i.e., periodic dielectric materials with photonic band gaps (PBGs).

**Fig. 6:**
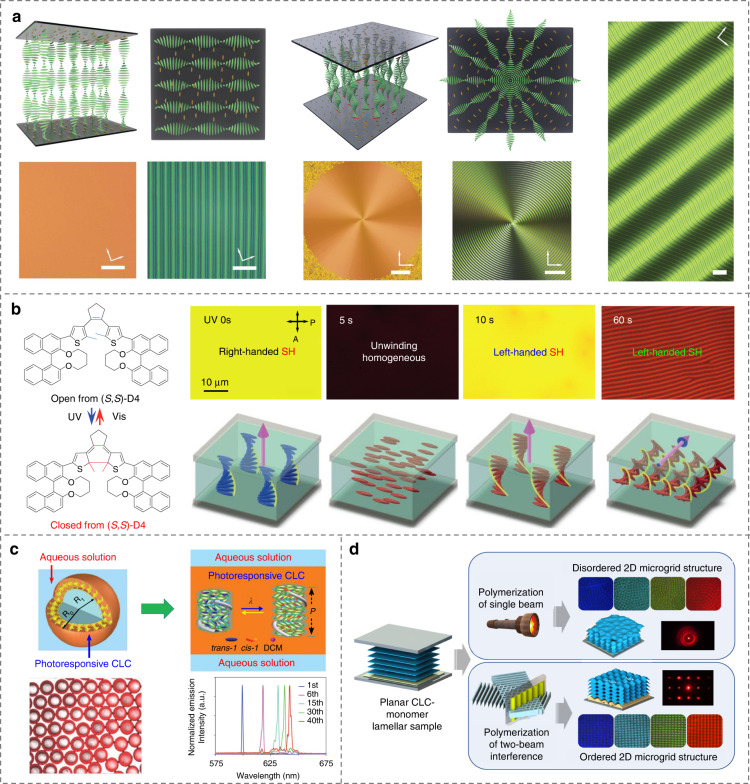
Creations and modulations of helical architectures in CLCs. **a** Photopatterned CLC helical superstructures^121^. Reproduced from ref. ^121^, with permission from Wiley-VCH. **b** 3D manipulation of the helical axis of CLCs by light^122^. Reproduced from ref. ^122^, with permission from Springer Nature: Nature. **c** Light-driven CLC microshells^124^. Reproduced from ref. ^124^, with permission from Wiley-VCH. **d** Photopolymerization-induced 2D helical deformations^125^. Adapted with permission from ref. ^125^. Copyright 2021 American Chemical Society

Sustaining efforts have been devoted to dynamically and multi-dimensionally controlling the CLC helical configurations. For example, the photoalignment technique was adopted to arbitrarily control the helical axis orientation of CLCs, allowing the creation of large-area, high-quality, and more complex fingerprint patterns, such as Achimedean spiral and wave-like grating^121^, Fig. 6a. Zheng et al.^122^ utilized a special dithienylcyclopentene-based molecular switch and achieved a 3D manipulation of the helical axis of CLC together with the inversion of the handedness solely by light, Fig. 6b. The tractable generation, modulation, and termination of zigzag patterns were also demonstrated^123^. Moreover, Chen et al.^124^ reported a photoresponsive microshell system with the tunable helical pitch enabled by a visible-light-driven chiral molecular switch, Fig. 6c. Jiang et al.^125^ reported 2D ordered microgrid chiral structures in a CLC reactive mixture due to the photopolymerization-induced periodic deformation, which looks like the Helfrich-Hurault undulation when the electric or magnetic field exceeds the threshold value^41,126^, Fig. 6d.

**Fig. 7:**
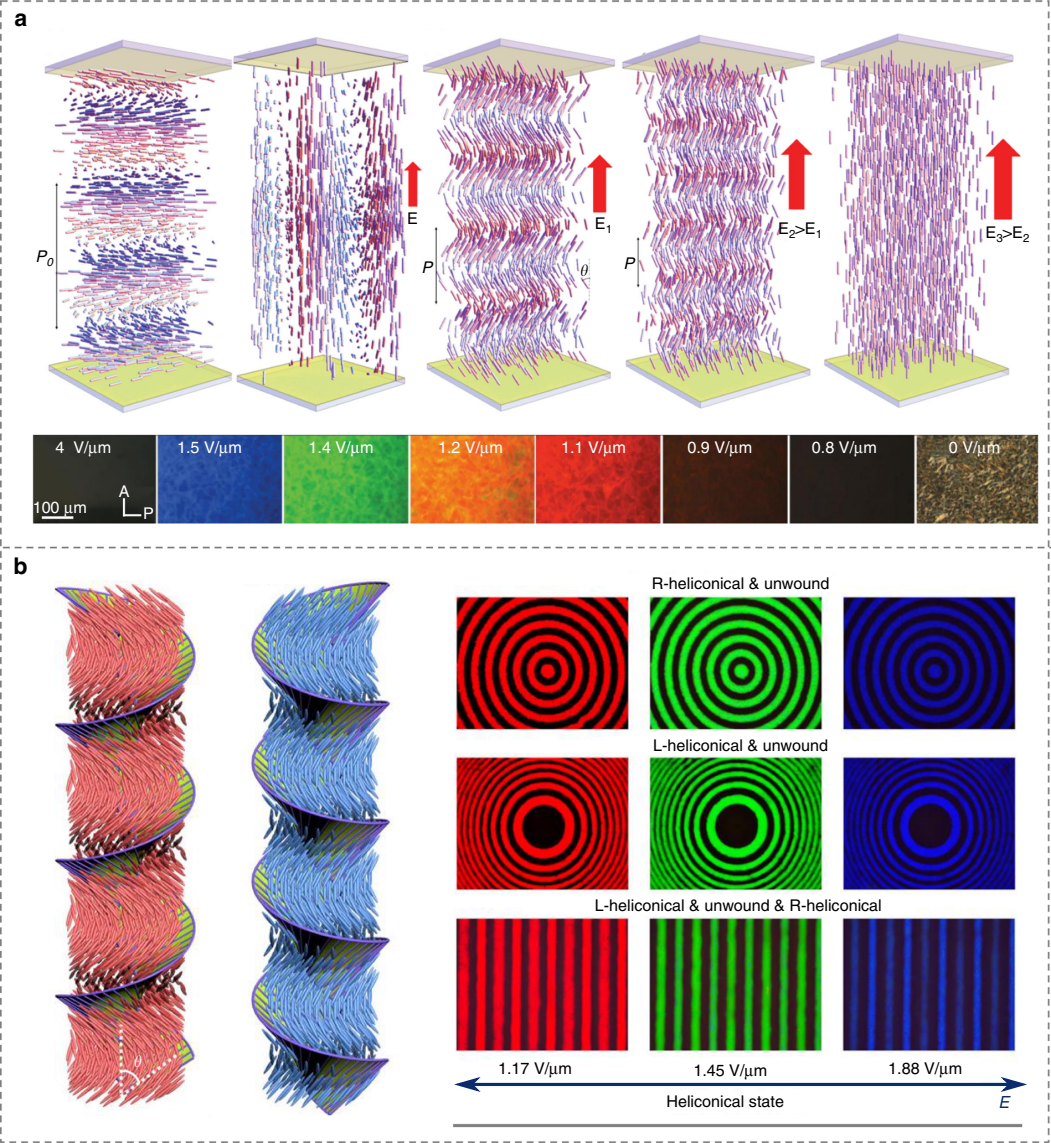
Dynamic tuning of oblique helicoidal architectures in CLCs. **a** Field-controlled oblique helicoidal CLC structure^38^. Reproduced from ref. ^38^, with permission from Wiley-VCH. **b** Electrically stimulated transformation of CLC superstructures^130^. Adapted from ref. ^130^, with permission from AAAS

Besides conventional cholesterics, CLCs with oblique helicoidal state were also disclosed^127^. The pitch of this state has been reported to be able to continuously tuned by external electric^38^, magnetic^128^ and light fields^129^. For instance, Xiang et al.^38^ produced electrically tunable selective reflection of light ranging from UV to visible and infrared regions by using the oblique helicoid CLC structure with low driving electric fields, Fig. 7a. Thanks to the preservation of the simple sinusoidal modulation of LC ordering at different fields, the oblique helicoidal CLC could achieve the maximum intensity of Bragg reflection, scattering, and resonances. The dual stimulation of light and electric field permits reversible and dynamic transformations between helicoidal and oblique helicoidal states along with the handedness inversion and dynamic PBG control^130^, Fig. 7b. These works broaden the scientific content of microscopic molecular self-assembly in soft chiral materials, which may inspire potential applications based on CLC superstructures.

### 3D cubic structures in blue phase LCs

Self-assembled blue phase LCs (BPLCs) are highly chiral states with unique complex 3D cubic lattices that cannot be artificially fabricated by micromachining^131,132^, Fig. 1. Three typical categories of BPLCs (BPI, BPII and BPIII), each of which has its unique structural characteristics, are determined by the strength of chiral interactions. BPI and BPII are composed of double-twist cylinders packed in body-centered and simple cubic lattices, respectively, and BPIII (foggy phase) possesses random and flexible structures, similar to the isotropic phase^133–135^. As the name indicates, one of the most noticeable properties of BPLCs is their selective reflection (like CLCs) with the reflected color largely depending on the helical pitch. In addition, the soft nature of LCs renders them highly responsive to external stimuli, such as electric field and light, resulting in tunable characteristics of BP-based optical performances^136^. The narrow temperature range of BPLCs is considered as an Achilles’ heel for practical applications. To overcome this shortcoming, polymer-stabilized BPLCs^131,137^, microstructure-stabilized BPLCs^138^, and new BPLC materials/composites^139–142^ were developed, following the pioneering work by Kikuchi^131^. Xiang and Lavrentovich^143^ further demonstrated a BP-templated soft material system to expand the temperature range.

**Fig. 8:**
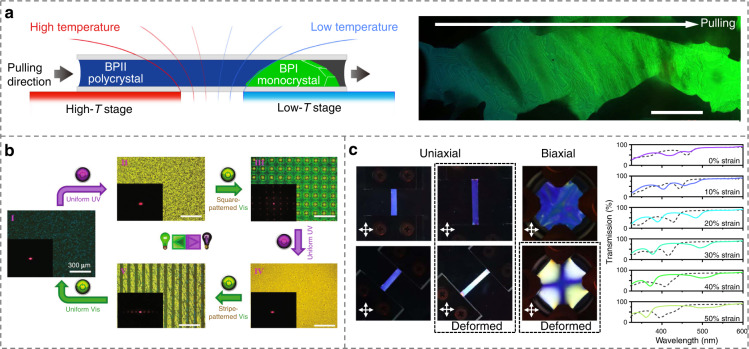
Creations and modulations of photonic structures in BPLCs. **a** Large single photonic crystal by a gradient-temperature scanning technique^144^. Adapted from ref. ^144^, with permission from Springer Nature: Nature communications. **b** Light-driven reconfiguration of biphasic micropatterns^146^. Reproduced from ref. ^146^, with permission from Wiley-VCH. **c** Mechanically deformed photonic structures in BPLCs^147^. Reproduced from ref. ^147^, with permission from Springer Nature: Nature communications

Recently, the monocrystalline alignment of BPLCs has received broad attention, because it avoids multicolored mosaic polycrystalline textures consisting of randomly distributed small platelet domains. Chen et al.^144^ reported large single photonic crystals in BPLCs based on a gradient-temperature scanning technique, Fig. 8a. These giant single crystals exhibited substantially sharp PBGs, long-range periodicity in all dimensions, and well-defined lattice orientation. The nucleation and growth of large uniform BPII single crystals with the domain size larger than 10 μm were developed on a chemically patterned substrate with alternative regions of different LC anchoring^43^. Moreover, Bukusoglu et al.^145^ studied the confinement and surface anchoring effects on the orientation of BPs, which provides a new tool to tailor the structure and optical properties of BP films.

By introducing a molecular switch functionalized nanocage, BPLCs are endowed with a reversible photoresponsive characteristic, which can be light-switched between the BPII and the cholesteric phases^146^. Consequently, well-defined biphasic micropatterns with both single soft cubic lattice and helical superstructures are disclosed, Fig. 8b. In addition, mechanical stimulation is another important manner to tune functional BPLC structures. Schlafmann et al.^147^ synthesized a fully solid BPLC elastomer that retains 3D nanostructures with dynamic reconfiguration upon photopolymerization. The remarkable tunabilities of the lattice constants and related optical performances of BPLC elastomers are validated through mechanical deformations as well as thermal and chemical stimulations, Fig. 8c. The electric field, environmental temperature, and humidity can alter the BP lattice orientation and transform the BP nanostructures as well, providing controllable on-demand optical properties^148–152^. The continuous progress of BPLC manipulation will advance the development of stimuli-responsive intelligent optical devices, such as high-performance 3D tunable lasers.

## Bio-based lyotropic LC architectures

Liquid-crystalline phase exists widely in biosystems through precisely controlled self-assembly, such as cell membranes, nucleic acids, proteins, polysaccharides and lipids. These bio-based LCs are also promising for soft matter photonics, which benefit the emergence of sustainable and biocompatible optical systems. Cellulose is among the most studied bio-LCs in this context owing to its availability in large quantities and outstanding optical/photonic material characteristics. It is a polysaccharide mainly derived from plants, fungi, and bacteria in the form of cellulose fibers, microfibers, and nanofibers. Over the past decades, great achievements have been made in the design and fabrication of optical micro- and nanostructures through the hierarchical manufacturing of celluloses to develop soft and smart optical devices for multiple high-tech applications, including smart displays, information processes, soft actuators, and smart windows^153–156^. In this section, we summarize the state-of-art photonic structure designs constructed from cellulose and their dynamic behaviors in response to environmental stimuli.

Cellulose is skillfully employed by plant kingdom to create unusual optical functions by assembling CLC nanostructures^153,157^. These photonic architectures have been an inspiration for the construction of artificial photonic materials based on cellulose that can mimic natural designs, properties, and functions^153,157^. Pure cellulose and cellulose derivatives, such as cellulose nanocrystals (CNCs), hydroxypropyl cellulose (HPC), and ethyl cellulose, can spontaneously self-assemble to generate CLCs^158–163^.

**Fig. 9:**
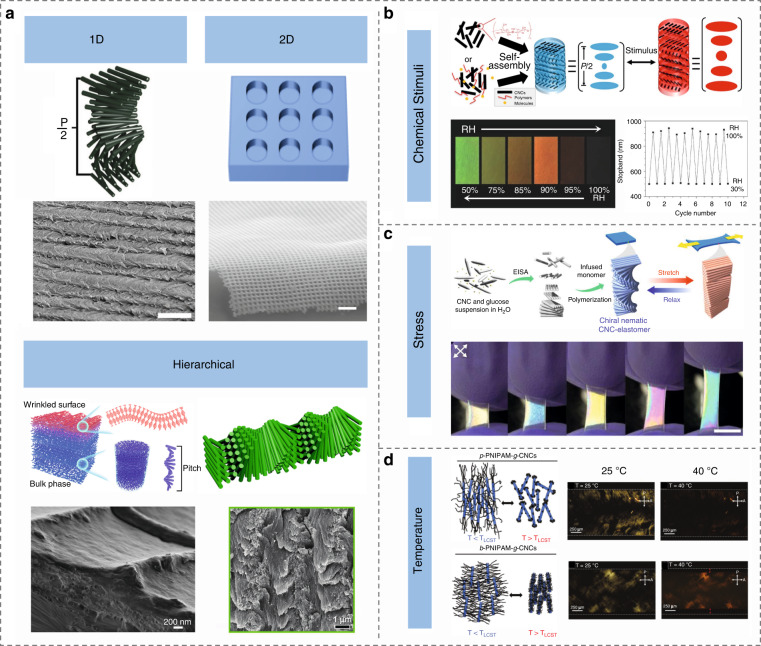
Cellulose-based representative photonic structures and the corresponding stimulus responsiveness. **a** Schematics (top rows) and images (bottom rows) of representative photonic architectures^163,170–172^. Images reproduced with permissions: 1D-helicoidal structure^163^, Wiley-VCH; 2D-grating structure^170^, Springer Nature: Nature Photonics; Hierarchical chiral nematic structure: left^171^, Elsevier; right^172^, Wiley-VCH. **b** Structure and structural color changes of CNCs under chemical and humidity stimulations^155,175^. Adapted with permission from ref. ^155^. Copyright 2020 American Chemical Society. Reproduced from ref. ^175^, with permission from Wiley-VCH. **c** Schematics and photographs showing the color changes of the CNC-elastomer composite under stretching^183^. Reproduced from ref. ^183^, with permission from Springer Nature: Nature Communications. **d** Temperature-responsive behavior and polarized optical microscopy images of patchy PNIPAM-grafted CNCs and “brush” PNIPAM-modified CNCs^192^. Reproduced from ref. ^192^, with permission from Wiley-VCH

CNC is a highly crystalline nanorod with a high aspect ratio that can spontaneously exhibit lyotropic LC behavior in a water suspension^164–167^. Such CLC structure can be preserved in solid films^164,168^. Because of the chiral interaction between nanorods, the CNC suspensions and films always self-assemble into left-handed CLCs to selectively reflect left-circularly polarized light^164–167^. The CLC structures of CNC films represent 1D photonic crystals that give iridescent colors, Fig. 9a. HPC is produced by the etherification of cellulose, which introduces hydroxypropyl groups onto the polymer chain. HPC has the same self-assembly performance as CNC, except that HPC has a right-handed chiral nematic structure, which is opposite to CNC^169^.

In addition to harnessing intrinsic 1D CLC structure for coloration, constructing nanoarchitectures on the surface of nanocellulose film is another effective approach. For example, 2D cellulose photonic crystals were fabricated by applying hot embossing or replica molding technique^170^, Fig. 9a. Both methods could produce highly-ordered periodic photonic structures to display characteristic iridescence. Hierarchical CLC structures were developed by shaping either surface topography or bulk periodicity on the microscale^171,172^. For instance, floral-mimetic hierarchically ordered photonic cellulose films that combine nanoscale CLC organization and microscale wrinkly surface topography were designed by leveraging soft nanoimprinting lithography^171^. The CNC nanorods close to the air-water interface can be freely assembled into CLC organization, while the CNC orientation near the template-CNC interface is anchored along the surface plane of its waved surface with the direction of the spiral axis remaining perpendicular to the undulating surface, Fig. 9a. In another scenario, CNC hydrogel containing vertically aligned uniform periodic structures was developed^172^. Such a thin hydrogel sheet contains CLC structures with helical axes parallel to the surface on the nanoscale and grating structures on the microscale, giving hierarchical signatures, Fig. 9a.

Cellulose-derived photonic structures are sensitive to various stimuli, such as humidity, solvents, gases, mechanical strain, and temperature, because of its large amount of hydroxyl groups and good compatibility with other materials^154,155^, enabling the establishment of dynamically responsive optical systems.

Cellulose is a hygroscopic material. Water molecules can strongly combine with hydroxyl groups in the amorphous region of CNCs or HPC, resulting in overall expansion and an increase in pitch^173,174^. Various compounds, such as polyethylene glycol (PEG)^175^, polyols^176^, polyacrylamide^177^, glucose^178^, N-methylmorpholine-N-oxide^179^, acrylamide^173^, have been added into the cellulose matrix to further improve its humidity responsiveness. For example, flexible CNC/PEG composite films with uniform and tunable structural colors were prepared as humidity sensors^175^, Fig. 9b. The addition of PEG allows an obvious improvement in the flexibility and the sensitivity of the photonic film to humidity. The composite film showed excellent cyclic stability and reversibility by constantly regulating humidity. Moreover, photonic cellulose films can also be adjusted to respond to organic solvents and gases^180–182^.

Mechanical stress, such as compression, shear, and stretching, is an effective method to manipulate optical properties of the cellulose films due to the rapid and reversible responsiveness, ease of handling, and controllable features. So far, several strategies such as coassembly with weakly interacting additives^178,183–187^, post processing^188^, or laminations^189,190^ have been pursued to generate flexible and mechanically responsive photonic films. Using a coassembly method, a uniform and stretchable CNC/elastomer composite was synthesized^183^, Fig. 9c. The resulting composite film can be stretched by over 900% and show reversible and rapid structural color changes. Mechanochromic HPC laminates were prepared by using large-scale, low-cost continuous coating and encapsulation^190^. The pressure response of HPC films can be quantified by optical analysis of pressure-induced color changes, enabling the recording of pressure distributions in real time such as a human footprint.

Polymers with thermal responsiveness are usually introduced into CNC matrix to enable thermally responsive chiral optical materials. Poly(N-isopropylacrylamide) (PNIPAM), known for its unique thermal and wet fracturing effects^191^, has been used to produce patchy PNIPAM-grafted CNCs with the aid of a surface-initiated atom transfer radical polymerization method^192^, Fig. 9d. The unique topological morphology design allows for an increase in translational and rotational degrees of freedom with the collapse of the PNIPAM chains. As such, its suspension exhibited optical anisotropic at 25 °C, but disappeared at 40 °C, Fig. 9d. This behavior is different from “brush” PNIPAM-modified CNCs, whose suspension exhibited birefringence at both 25 °C and 40 °C, Fig. 9d.

## Applications

### Smart displays

A primary goal of displays is to create smart devices which can adaptively respond to various external stimuli, such as light, electricity, and force, delivering real-time desired information. Structural colors, which rely on the meticulous design of microstructural architectures to obtain colorful characteristics without pigments or dyes, show promising potentials for applications in displays, decoration, and anti-counterfeiting^193,194^, due to their distinctive features of environmental friendliness and high stability, and capacity to produce brilliant, fading-resistant, tunable, and high-resolution colors. Here, we focus on the stimuli-adaptive color-tuning display systems based on structural colors.

**Fig. 10:**
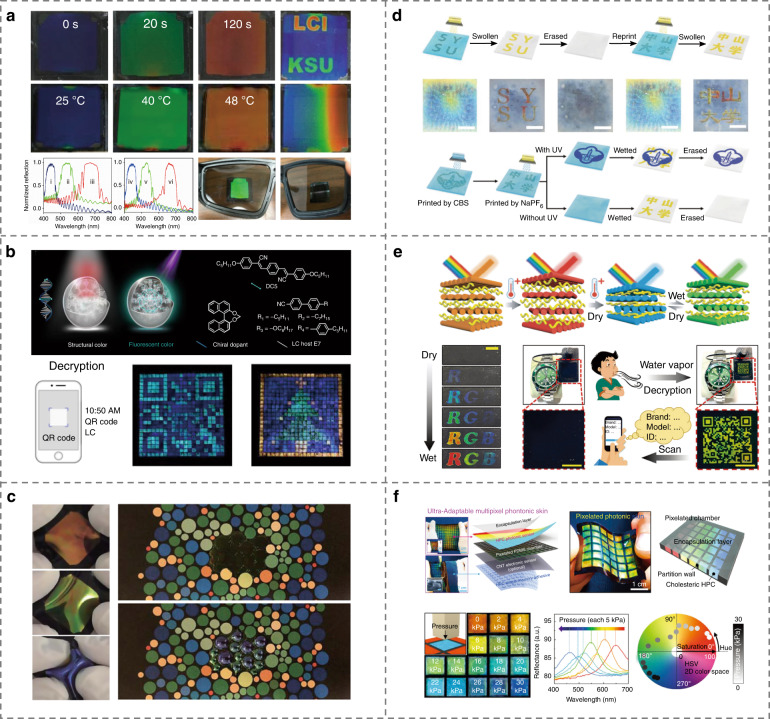
LC-based smart displays and information process. **a** Full-color reflected display by photoresponsive CLCs^195^. Reproduced from ref. ^195^, with permission from Wiley-VCH. **b** Two-tone microdroplet based geminate labels^197^. Adapted from ref. ^197^, with permission from Springer Nature: Nature Communications. **c** Pneumatic actuated divergent colorations in CLC elastomers^199^. Reproduced from ref. ^199^, with permission from Springer Nature: Nature Materials. **d** Reversible writing/erasing process of patterns on the CNC/polycation photonic paper^203^. Reproduced from ref. ^203^, with permission from Wiley-VCH. **e** Multicolor pattern and QR code derived from HPC-acrylamide composite^173^. Reproduced from ref. ^173^, with permission from Wiley-VCH. **f** HPC-based photonic skin^204^. Reproduced from ref. ^204^, with permission from Wiley-VCH

The way to remotely, spatially, and temporally control the structural color from CLCs has attracted significant attention. Recently, Wang et al.^195^ achieved reversibly light-activated structural colors across the whole visible spectrum through the photoisomerization of a halogen-bonded axially chiral switch, which is an important step toward smart photodisplay devices, Fig. 10a. By employing a visible-light-driven chiral fluorescent molecular switch, rewritable multimodal CLC fluorescence/reflection display devices were realized by Li et al.^115^. Qin et al.^196^ proposed a new strategy of reflective displays with light-driven black ground and RGB structural colors. In addition, geminate security labels have been realized by patterning two-tone CLC microdroplets with both the structural and fluorescent colors, Fig. 10b, which opened a new avenue for anti-counterfeiting technologies^197^. In addition, unique optical materials of oblique helicoidal CLCs are also appealing candidates for smart displays^129,198^, which possess both twist and bend LC orientations, and are highly sensitive to the electric and light fields, resulting in a feasible tunability of the structural color consequently^129^.

CLC elastomers serve as a structural-color-changing platform as well, which not only preserve the self-organization of LCs but also enable a strain deformation^199–201^. Kim et al.^199^ proposed twin-layer pneumatically inflating thin membranes composed of highly stretchable CLC elastomers and PDMS film to demonstrate compact pixelized structural colors with broadband spectral tunability, Fig. 10c. The different transverse deformations via pneumatic actuation lead to divergent colorations, which originates from the anisotropic elasticity induced large Poisson’s ratios of LCs. Schmidtke et al.^202^ found that the actuation of biaxial stress results in substantially enhanced photonic properties of free-standing CLC elastomer coatings, allowing potential applications in tunable optical filters.

The capacity to create unique chiral optical properties, display vivid structural colors, and to respond rapidly and reversibly to a variety of stimuli makes photonic cellulose films potentially viable for smart display, information encryption, and ant-counterfeiting applications^154^. As an example, a re-printable photonic paper was prepared by incorporating chiral nematic CNCs into a chemically-crosslinked polycation^203^. The film showed controllable wettability via anion exchange, resulting in extremely low color contrast in the dry state but high contrast in the wet state, which enables reversible display and hiding of the encoded information, Fig. 10d. In another study, a series of structural color materials with multiple dynamic photonic responsiveness and high-resolution patterns were developed by mixing cellulose molecules with acrylamide monomers^173^. By synergistically utilizing dual-responsive behavior to humidity and UV light, a multicolor pattern and a quick response (QR) code were demonstrated, Fig. 10e. Flexible cellulose photonic materials that are capable of significantly simplifying device construction, allowing real-time stimulation visualization, and readily detecting mechanical and physical signals have shown the potential as “photonic skins” to replace bulky and rigid electronic devices.

By integrating the shape memory and self-assembly characteristics of HPC simultaneously into a multi-layer flexible film structure, an ultra-adaptive and stably wearable pixelated photonic skin that can be used for precise monitoring of human motion and structural health of buildings and bridges was developed^204^, Fig. 10f. Such pixelated photonic device allows for a vivid color response by applying different mechanical stimuli to it. A clear color transition from red to blue was observed when the applied pressure is increased, Fig. 10f.

### Optical imaging

Optical lens is a common but indispensable optical element in diverse imaging areas, such as telescopes, binoculars, and cameras^205–207^. Traditional optical lens mainly depends on the regulation of dynamic phase based on the isotropic medium, which hinders the lightweight, miniaturization, and integration. The Pancharatnam-Berry (PB) phase is discovered by S. Pancharatnam (1956)^208^, and later generalized by M. Berry (1984)^209^, which is a geometric phase associated with the polarization of light^210^. Through rationally designing the director field of LCs, LC imaging devices can be developed based on PB phase, which show non-negligible merits of planar and ultra-thin configurations, tunable characteristics, and especially polarization slectivity^211–214^.

**Fig. 11:**
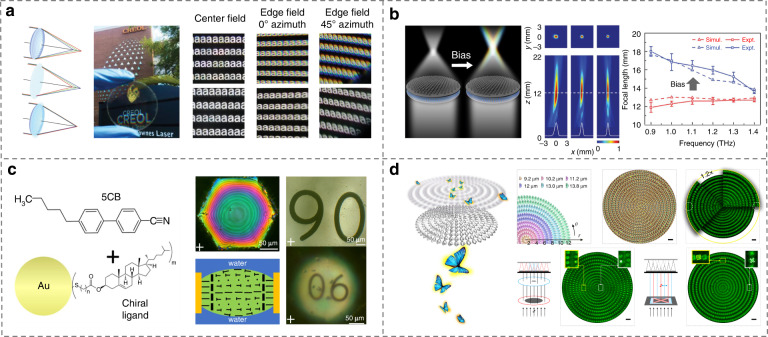
LC microlenses for optical imaging. **a** Hybrid lens by combining the Fresnel lens and PB lens effects^216^. Reproduced from ref. ^216^, with permission from Wiley-VCH. **b** LC based tunable chromatic aberration metalens for terahertz imaging^217^. Reproduced from ref. ^217^, with permission from SPIE and CLP. **c** Au nanoparticle doped CLC lens immersed in water^220^. Adapted with permission from ref. ^220^. Copyright 2021 American Chemical Society. **d** 4D visual imaging by SLC topological defects^98^. Adapted with permission from ref. ^98^. Copyright 2019 American Chemical Society

Recently, High-quality reflective polymeric CLC PB lenses were reported with a diameter D = 2.45 cm and low *f*-numbers (*f*/2, *f*/0.9, *f*/0.45, *f*/0.33) at 550 nm^215^, which can be converging or diverging, depending on the handedness and direction of the incident light. Zhan et al.^216^ presented large-scale, cost-effective, and ultra-broadband PB lenses with structured LC polymers, which overcame the critical issue of chromatic aberration originating from the optical dispersions of materials, Fig. 11a. Shen et al.^217^ proposed a strategy to create tunable microlenses operating in THz region by delicately integrating metasurface and LCs, Fig. 11b. They further demonstrated switchable chromatic aberration by applying a bias voltage for different purposes. CLC is also a kind of popular soft material in lens fabrications^218–220^. A PB phase lenticular microlens with a polarization-dependent focal length by immersing chiral gold nanoparticles doped CLCs in water was reported by Perera et al.^220^, Fig. 11c.

Polarization imaging, especially four-dimensional visual imaging including 1D polarization and 3D space information of the target, is a special and promising technology for future optical imaging^98,221–224^. Recently, Ma et al.^98^ proposed an efficient approach based on a well-designed asymmetric topological microlens array for the four-dimensional visual imaging by a single snapshot, Fig. 11d. The demultiplexing of both the depth and polarization information carried by the targets was demonstrated. The above studies show the superiority of LCs in the accurate control and manipulation of the light propagation, which are expected to innovate existing imaging technologies for the needs of contemporary science and technology.

### Light field modulation

**Fig. 12:**
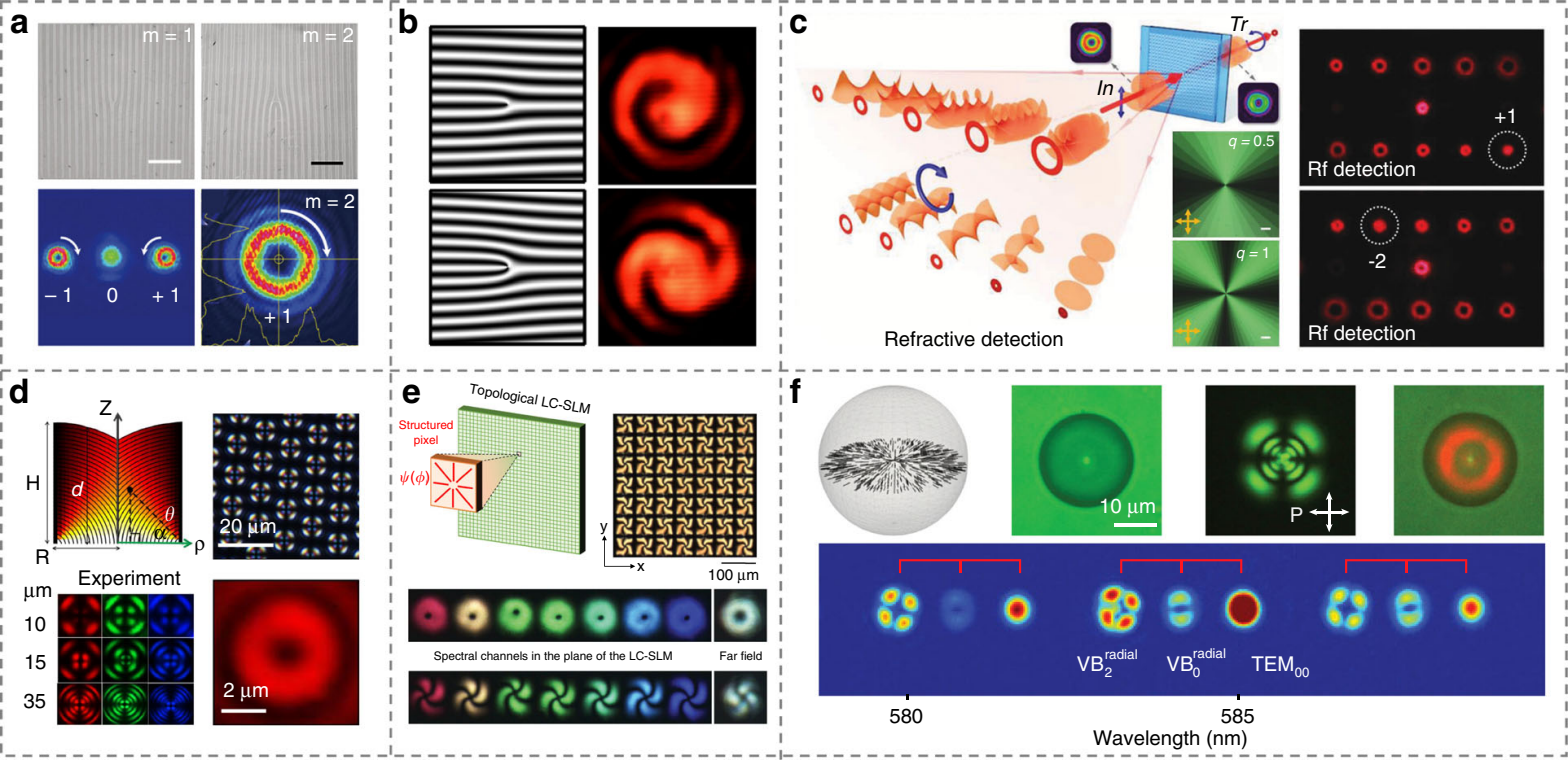
LCs for light field modulation devices. **a** Transmissive vortex beams generated by diffraction fork gratings in NLCs^67^. Reproduced from ref. ^67^, with permission from Wiley-VCH. **b** Reflective vortex beams generated by diffraction fork gratings in CLCs^240^. Reproduced with permission from ref. ^240^. Copyright 2016, American Physical Society. **c** Detection of OAM modes by CLC Dammann fork gratings^241^. Reproduced from ref. ^241^, with permission from Wiley-VCH. **d** Large area optical vortex arrays based on FCDs in SLC^99^. Adapted with permission from ref. ^99^ © The Optical Society. **e** Spatial and temporal modulation of multispectral light fields by a programmable LC SLM^245^. Reproduced with permission from ref. ^245^. Copyright 2018, American Physical Society. **f** Tunable structured light microlasers based on topological LC superstructures^247^. Reproduced from ref. ^247^, with permission from National Academy of Sciences

Thanks to the high birefringence, self-assembled superstructure, huge sensitivity to external fields, and easier manipulation of optical axis distributions that differ from solid crystals^120^, LCs come into prominence in the field of light field modulation^225–228^, and present dynamic optical functions just like or even beyond metasurfaces ranging from visible to terahertz regions^217,229,230^. Vortex beam is one of the most popular and versatile beams^231,232^, with wide applications in optical tweezers^233^, optical communication^234^, quantum computation^235^, and coronagraph^236^. Till now, plentiful of optical schemes have been proposed to generate vortex beams^237–239^, such as fork gratings, q-plates, spin-orbit coupling, spatial light modulators (SLMs), and deformable mirrors, etc. Among them, LC-based light field modulation devices occupy an important position. For instance, Wei et al.^67^ demonstrated LC fork gratings for the vortex beam generation by using a DMD-based dynamic mask photopatterning system, Fig. 12a. Kobashi et al.^240^ demonstrated the efficient and polychromatic generation of broadband optical vortices by creatively adopting CLC fork gratings, Fig. 12b. Moreover, LCs are also effective tools for the detection of orbital angular momentum (OAM) modes, especially in mode-division multiplexing. Chen et al.^241^ further introduced a concept of digitalized chiral superstructures and produced a Dammann fork grating for simultaneous detection of multiplexed optical vortex beams without the mode crosstalk or distortion, Fig. 12c. By using rationally photoaligned CLCs, detections including vector beam and hybrid OAM modes were all successfully realized, which greatly expands the role of LCs in optical communications.

LC defects can also be exploited for the generation and modulation of the optical field^242,243^. Voloschenko et al.^244^ firstly clarified vortex beams generated by dislocations in CLC finger structures. Son et al.^99^ generated large area optical vortex arrays based on micron-sized FCDs, Fig. 12d. Each FCD was able to produce an optical vortex with topological charge confirmed by interference pattern. Nassiri and Brasselet^245^ manufactured a topological LC SLM, making it possible to control OAM states on multispectral channels, Fig. 12e. Polychromatic superposition of OAM states and 4D optical pulse shaping were realized by topological SLM, providing a convenient platform for both spatial and temporal manipulation of the light field. LC droplets are proposed as another tool to generate structured light^246^. Recently, Papič et al.^247^ demonstrated a tunable structured light microlaser based on topological LC superstructures in a Fabry-Perot microcavity, Fig. 12f. Different modes of vector laser beams were effectively emitted from a radial nematic droplet. By elaborately designing the topological LC superstructures, more structured light lasers were shown, enabling tunable and compact photonic devices. Besides, there are also lots of works dealing with spatiotemporal optical vortex^248^, spin-split photon mode^249^ and 3D spiral optical fields^239^ based on liquid crystalline materials, which may provide a satisfactory platform for tough requirements in cutting-edge photonics.

### Soft actuators

Soft actuators have been a hot research theme for decades due to their significant flexibility in operations. LC-based materials are particularly encouraging, which can macroscopically deform in response to various external stimuli (light^250–253^, heat^254,255^, electric^37,256,257^ and magnetic^258^ fields), instead of using mechanical force to generate dynamic change. In addition, the facile engineering of LC microstructures adds another degree of freedom to control the shape change and motion of soft actuator^40,253,259^, enabling a series of complex tasks, such as oscillation, rotating, rolling, turning, twisting as well as their combinations, which are expected to replace current machinery parts.

**Fig. 13:**
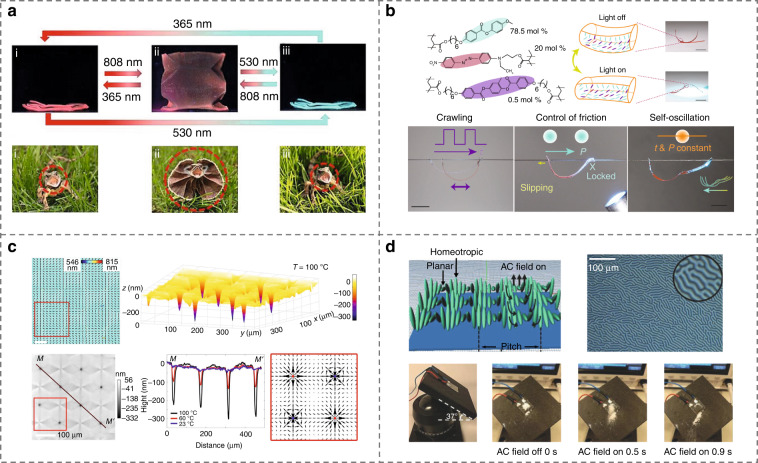
Polymerized LC films for soft actuators. **a** Synergistic photochromic luminescence and programmable soft actuators based on LC networks^251^. Reproduced from ref. ^251^, with permission of Wiley-VCH. **b** Light-activated LC actuators climbing on human hairs^261^. Reproduced from ref. ^261^, with permission from Wiley-VCH. **c** LC elastomer coatings with programmed surface topographies^262^. Adapted from ref. ^262^, with permission from Springer Nature: Nature Communications. **d** Electrically driven oscillating fingerprints for dust control^257^. Reproduced from ref. ^257^, with permission from Wiley-VCH

Photoresponsive LC elastomers with light-driven flexible actuations have gained significant interest^250–252,260^. To generate highly programmable soft actuators, Huang et al.^251^ integrated tunable fluorophores into LC elastomers. The combination of strong fluorescent emission and reversibly photoisomerization-induced deformation was utilized to mimic multiple biological functionalities, such as the shape morphing and discoloration behaviors of *frillneck lizard*s, Fig. 13a. Cheng et al.^261^ demonstrated the light-controlled friction and locomotion of a centimeter-long polymer stripe under a constrained condition of a human hair, Fig. 13b. The friction conditions of both the hair surface and the asymmetric actuator geometry, together with the photo-actuated LC deformation, lead to versatile directional locomotion.

Moreover, Babakhanov et al.^262^ fabricated a series of thermoresponsive LC elastomer coatings with preprogrammed topological molecular orientations, Fig. 13c. The dynamically thermal control of the surface topographies allows for particle rearrangements^262,263^. Oscillating CLC fingerprints were also achieved to wipe away dust by electrically modulating the topographic corrugation, Fig. 13d. Feng et al.^257^.

**Fig. 14:**
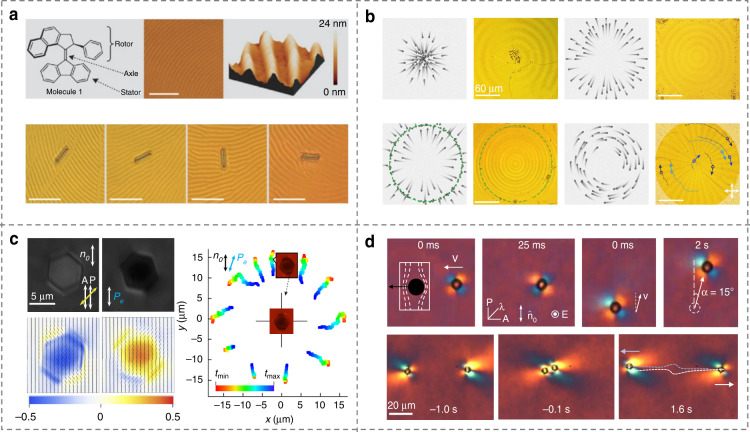
LC-based soft actuators. **a** Rotary manipulators based on photoresponsive CLC fingerprints^264^. Reproduced from ref. ^264^, with permission from Springer Nature: Nature. **b** Programmable self-propelling actuators enabled by a dynamic helical medium^30^. Adapted from ref. ^30^, with permission from AAAS. **c** Self-assembled reconfigurable colloidal monopoles^266^. Adapted from ref. ^266^, with permission from Springer Nature: Nature. **d** Particle manipulation based on soliton-induced electrophoresis^75^. Adapted with permission from ref. ^75^. Copyright 2020, American Physical Society

Besides polymerized LC actuators, small-molecular LCs with fluidic elastic properties can be used as functional actuators as well^73,122,264,265^. In 2006, Eelkema et al.^264^ reported a collectively rotational CLC fingerprint system that rotated microscopic-scale objects by introducing a light-driven rotary nanomachine, Fig. 14a. Ma et al.^30^ achieved programmable self-propelling actuators that could massively transport microparticles in customized trajectories by elaborately designing the self-organized microstructure-engaged CLC system, Fig. 14b. Yuan et al.^266^ demonstrated the reconfigurable colloidal assembly based on optically switchable signs and amplitudes of the interactions of elastic colloidal monopoles, Fig. 14c. They also developed self-assembled LC colloidal nanomotors, enabling the unidirectional particle to spin with light-controlled handedness and frequency^267^. In addition, the dynamic manipulation of soliton-dressed spherical particles is also accomplished by Li et al.^75^ based on the alternating current electrophoresis in NLCs, Fig. 14d.

**Fig. 15:**
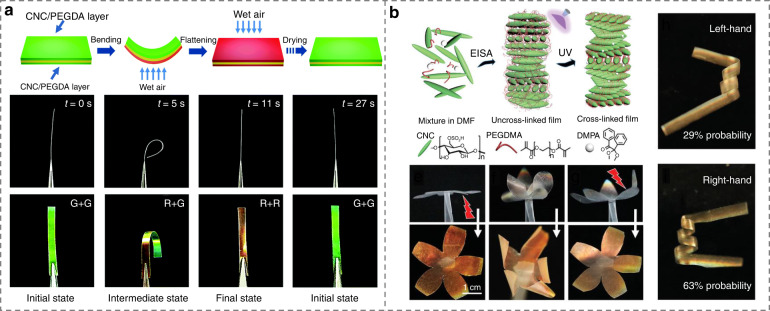
bio-based LC soft actuators. **a** Deformation and color change of sandwich CNC film driven by humidity^275^. Reproduced from ref. ^275^, with permission from The Royal Society of Chemistry. **b** Moisture-driven actuation and complex deformation behaviors of the CNC-poly(ethylene glycol) dimethacrylate composite^42^. Reproduced from ref. ^42^, with permission from Wiley-VCH

Soft actuators based on photonic nanocelluloses have drawn great attention because of their superior humidity response, mechanical flexibility, color sensing, and biocompatibility, opening a sustainable avenue in visual mechanical sensors, wearable photonics, smart bionic actuators, and intelligent robots^42,268–275^. For example, by mimicking the shell structure of scarab beetles, a photonic actuator based on CNCs was developed^275^. The composite films were prepared by sandwiching a uniaxial orientation polymer layer between two flexible CNC layers. The increase of environment humidity leads to the bend of the layered photonic films away from the wet air and consequently the change of structural color, Fig. 15a. Such photonic actuators showed excellent dynamic reversibility upon cyclic humidity change. More recently, mechanically flexible, deformable, and optically tunable composites were fabricated by assembling CNC with polyethylene glycol dimethacrylate (PEGMA) monomer^42^. Such photonic films exhibited sensitive and reversible moisture-driven actuation behavior and a variety of complex 3D deformation modes, accompanied by the variation of color appearances, Fig. 15b.

### Smart windows

As one of the least energy-efficient components in buildings, there is burgeoning interest in developing smart windows with the capability to dynamically control the transmittance of sunlight, lending benefits including energy efficiency, architectural beauty, eye protection, and privacy protection^276–278^. Thanks to the multiple stimuli-responsiveness, LCs as well as their composites are promising candidates for smart windows^279,280^.

**Fig. 16:**
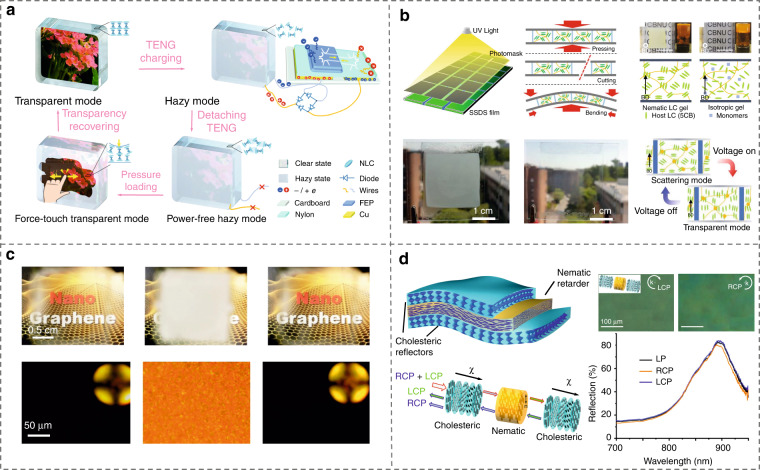
Smart window applications. **a** A self-powered hybrid switchable solar window based on CLCs and a sliding triboelectric nanogenerator^279^. Adapted from ref. ^279^, with permission from Elsevier. **b** Robust LC smart windows with low voltage switching^283^. Reproduced from ref. ^283^, with permission from Wiley-VCH. **c** Smart windows based on LC/graphene composites^285^. Reproduced from ref. ^285^, with permission from Elsevier. **d** CNC-based tunable photonic reflectors^287^. Adapted with permission from ref. ^287^. Copyright 2018 American Chemical Society

The most well-known LC smart windows are based on polymer-dispersed LC (PDLC)^281^, where LC molecules are in the form of droplets dispersed in polymers, with fast response ability. To maintain an energy-efficient window view, self-powered smart windows are highly pursued^279,282^. Murray et al.^282^ developed a self-powered hybrid switchable solar window by combining the PDLC system with a semiconducting absorber, enabling electrically controllable light trapping and on-demand power generation throughout the day. Another fully self-powered and ultra-stable smart window was proposed based on elaborately fabricated CLCs and the triboelectric nanogenerator^279^, Fig. 16a. Thus, the window can be facilely driven between transparency and haziness by instantaneous mechanical stimuli. Recently, Yoon et al.^283^ generated a robust LC smart window by a single-step dual-stabilization of LCs mixed with photoresponsive dopants and monomers, Fig. 16b. This smart window based on physical gels and polymer chambers exhibits fast response and low voltage actuation. Harnessing the beneficial features of nanomaterials into host materials provides a facile path to endow smart windows with multi-responsive and multi-functional capabilities^284^. Wang et al.^285^ achieved a homogeneous dispersion of 2D materials into chiral LC superstructures by elaborately synthesizing mesogen-functionalized graphene, which enables multi-responsive smart window systems, Fig. 16c. Energy-efficient smart window applications were also demonstrated by using a roll-to-roll process to fabricate LC composites containing tungsten bronze nanorods^286^. They can reversibly switch the transmittance of light by temperature, electric field, and near-infrared light, showing advantages of wide temperature range, high flexibility, robust mechanical strength, long-term stability, and large-area processability.

In addition to typical thermotropic LCs, cellulose-based LC films have also been utilized as tunable reflectors for thermal management^156^. For example, De La Cruz et al.^287^ designed a photonic composite film consisting of two cholesteric CNC reflectors sandwiching a nematic-like CNC retarder, Fig. 16d. This sandwich structure possesses a polarization-independent reflection (i.e. both left-handed and right-handed circularly light can be effectively reflected), much higher reflection than a single cholesteric-like film, and tunable reflection from the visible to near-infrared ranges of the optical spectrum. The composite could achieve high reflectivity in the near-infrared range while maintaining high transmittance in the visible spectral regime, making it suitable for smart window applications. Besides, smart windows were also designed based on a biopolymer-stabilized LC system composed of a CNC-based network and a nematic LC^288^. This system shows rapid voltage-off response time, good voltage-driven contrast between the scattering and transparent states, and a high haze factor.

## Conclusion and prospects

Soft matter photonics (we refer to it here as “Soft Mattonics”) is a burgeoning area of research and has attracted much attention in recent years. By combining “top-down” manufacturing technique with “bottom-up” self-assembly process of LCs, one can design and fabricate hierarchical superstructures with multiple degrees of freedom, which makes them splendid candidates for soft and smart photonics. In this article, we have highlighted recent studies focusing on the creation, manipulation, and application of self-assembled optical architectures based on typical thermotropic LCs (NLCs, SLCs, CLCs, and BPLCs) and bio-based lyotropic LCs (CNCs and HPCs). These soft materials show distinguished optical and responsive properties that are employed for different applications. For examples, NLCs exhibit long-range orientational ordering and the LC director distribution can be collectively modified by external fields, resulting in a large modulation of the optical phase and consequently the light transmittance. SLCs with highly ordered structures present both the orientational order and the positional order. They can self-organize into various topological defects with functionalities of micro-imaging, particle manipulating, beam steering, etc. CLCs with 1D periodic helical structures possess a PBG caused by Bragg reflection, which endows them with promising applications related to the control of specific polarization/wavelength dependent functions. Self-organized double-twisted BPLCs are 3D photonic materials with high chirality and degree of freedom in developing optical and photonic devices. In addition, the capability to construct photonic structures with different topologies and topographies provides complex, tunable, and multiple functionalities to these soft-matter-based optical platforms. Corresponding applications have been demonstrated, for instance, smart display, optical imaging, and light field modulation devices.

Although continuous and great progress has been made, related research in Soft Mattonics is still at its preliminary stage. It remains strong challenges for large-scale production and processing of these soft optical devices, owing to the difficulties in achieving uniformity of soft matter films, precise patterning over large areas, and stability of the systems. Realizing optimized “structure-property-function” relationships through efficient manufacturing technologies is still difficult. In addition, the seamless integration of soft-matter optical materials (as we described here) with existing optical components is an open challenge. Therefore, much effort needs to be devoted to exploring new manufacturing technologies to mutually optimize the correlations of materials, structures, and functions.

It is anticipated that self-assembled LC architectures with merits of easy fabrication, fine tunability, high flexibility, and remarkable stimuli-responsiveness would play important roles in the prosperous development of optoelectronics, optics, and photonics. More appealing soft architectures can be further expected in newly discovered LC phases. For example, Noel Clark group recently discovered ferroelectric nematic liquid crystalline phase with huge *ε* and high fluidity, which may open a band-new door for the nontrivial architectures with an exciting future^289^. In addition, Soft Mattonics with new optical functions would be worth expecting by combining LCs with other soft materials. For instance, silk protein, which is a natural structural protein that is mainly spun by spiders and silkworms, is an excellent candidate for soft optical materials owing to its capacity to develop a wide variety of photonic architectures that generate an optical response as a result of interaction between light and the nanostructure in which silk is molded, and to create flexible, tunable, complex, and multifunctional optical platforms^290–295^. The development of LC-silk photonic composites would provide expanded optical utility by leveraging their functional interplay and further extend the applications of LC devices towards the interface between optical technologies and biological world. By integrating LCs with cutting-edge electronic and robotic systems, multifunctional devices and advanced optical systems with desirable adaptive and active performances can be energetically anticipated. Further exploration in such thriving topic would not only broaden the knowledge of Soft Mattonics but also encourage multidisciplinary research from specialists across different disciplines and promote diverse soft and smart photonic applications.

## References

[1] De Gennes, P. G. & Prost, J. The physics of liquid crystals. 2nd edn. (Oxford: Oxford University Press, 1995).

[2] Kléman, M. & Lavrentovich, O. D. Soft matter physics: an introduction. (New York: Springer, 2003).

[3] Fernández-Rico C (2020). Shaping colloidal bananas to reveal biaxial, splay-bend nematic, and smectic phases. Science.

[4] Bisoyi HK, Li Q (2022). Liquid crystals: Versatile self-organized smart soft materials. Chem. Rev..

[5] Liu PW (2022). Biomimetic confined self-assembly of chitin nanocrystals. Nano Today.

[6] Yang, D. K. & Wu, S. T. Fundamentals of liquid crystal devices. 2nd edn. (Hoboken: John Wiley & Sons, 2014).

[7] Whitesides GM, Grzybowski B (2002). Self-assembly at all scales. Science.

[8] Scacchi A (2021). Self-assembly in soft matter with multiple length scales. Phys. Rev. Res..

[9] Hough LE (2009). Helical nanofilament phases. Science.

[10] Wang XG (2016). Topological defects in liquid crystals as templates for molecular self-assembly. Nat. Mater..

[11] Chen Q, Bae SC, Granick S (2011). Directed self-assembly of a colloidal kagome lattice. Nature.

[12] Piccardi A (2014). Power-controlled transition from standard to negative refraction in reorientational soft matter. Nat. Commun..

[13] Gibaud T (2012). Reconfigurable self-assembly through chiral control of interfacial tension. Nature.

[14] Teyssier J (2015). Photonic crystals cause active colour change in chameleons. Nat. Commun..

[15] Chou HH (2015). A chameleon-inspired stretchable electronic skin with interactive colour changing controlled by tactile sensing. Nat. Commun..

[16] Yu YD (2020). Chameleon-inspired stress-responsive multicolored ultratough films. ACS Appl. Mater. Interfaces.

[17] Cao Y (2020). Deciphering chiral structures in soft materials *via* resonant soft and tender X-ray scattering. Giant.

[18] Isapour G, Lattuada M (2018). Bioinspired stimuli-responsive color-changing systems. Adv. Mater..

[19] Zhang H (2019). Programmable responsive hydrogels inspired by classical conditioning algorithm. Nat. Commun..

[20] Zeng H (2020). Associative learning by classical conditioning in liquid crystal network actuators. Matter.

[21] Lv JA (2016). Photocontrol of fluid slugs in liquid crystal polymer microactuators. Nature.

[22] Rich SI, Wood RJ, Majidi C (2018). Untethered soft robotics. Nat. Electron..

[23] Shimizu T, Ding W, Kameta N (2020). Soft-matter nanotubes: A platform for diverse functions and applications. Chem. Rev..

[24] Poy G (2020). Chirality-enhanced periodic self-focusing of light in soft birefringent media. Phys. Rev. Lett..

[25] Dierking, I. Textures of Liquid Crystals. (Weinheim: John Wiley & Sons, 2003).

[26] Ackerman PJ, Smalyukh II (2017). Static three-dimensional topological solitons in fluid chiral ferromagnets and colloids. Nat. Mater..

[27] Xia Y (2018). Thickness-independent capacitance of vertically aligned liquid-crystalline MXenes. Nature.

[28] Xiong J (2022). Holo-imprinting polarization optics with a reflective liquid crystal hologram template. Light Sci. Appl..

[29] Badloe T (2022). Liquid crystal-powered Mie resonators for electrically tunable photorealistic color gradients and dark blacks. Light Sci. Appl..

[30] Ma LL (2021). Programmable self-propelling actuators enabled by a dynamic helical medium. Sci. Adv..

[31] Ihn KJ (1992). Observations of the liquid-crystal analog of the Abrikosov phase. Science.

[32] Mitov M (2017). Cholesteric liquid crystals in living matter. Soft Matter.

[33] Nakata M (2007). End-to-end stacking and liquid crystal condensation of 6- to 20-base pair DNA duplexes. Science.

[34] Wang L, Urbas AM, Li Q (2020). Nature-inspired emerging chiral liquid crystal nanostructures: From molecular self-assembly to DNA mesophase and nanocolloids. Adv. Mater..

[35] Wang Y (2015). Room temperature heliconical twist-bend nematic liquid crystal. CrystEngComm.

[36] Bisoyi HK, Li Q (2014). Light-directing chiral liquid crystal nanostructures: From 1D to 3D. Acc. Chem. Res..

[37] He Q (2019). Electrically controlled liquid crystal elastomer-based soft tubular actuator with multimodal actuation. Sci. Adv..

[38] Xiang J (2015). Electrically tunable selective reflection of light from ultraviolet to visible and infrared by heliconical cholesterics. Adv. Mater..

[39] Bisoyi HK, Li Q (2016). Light-driven liquid crystalline materials: From photo-induced phase transitions and property modulations to applications. Chem. Rev..

[40] Shahsavan H (2020). Bioinspired underwater locomotion of light-driven liquid crystal gels. Proc. Natl Acad. Sci. USA.

[41] Hurault JP (1973). Static distortions of a cholesteric planar structure induced by magnetic or ac electric fields. J. Chem. Phys..

[42] Ge W (2022). Highly tough, stretchable, and solvent-resistant cellulose nanocrystal photonic films for mechanochromism and actuator properties. Small.

[43] Li X (2021). Nucleation and growth of blue phase liquid crystals on chemically-patterned surfaces: A surface anchoring assisted blue phase correlation length. Mol. Syst. Des. Eng..

[44] Zhang H (2020). Azobenzene sulphonic dye photoalignment as a means to fabricate liquid crystalline conjugated polymer chain-orientation-based optical structures. Adv. Optical Mater..

[45] Yeh, P. & Gu, C. Optics of liquid crystal displays. (New York: John Wiley & Sons, 1999).

[46] Goodby, J. W. et al. Handbook of liquid crystals. 2nd edn. (Weinheim: John Wiley & Sons, 2014).

[47] Xiong J, Wu ST (2021). Planar liquid crystal polarization optics for augmented reality and virtual reality: From fundamentals to applications. eLight.

[48] Honda M, Seki T, Takeoka Y (2009). Dual tuning of the photonic band-gap structure in soft photonic crystals. Adv. Mater..

[49] Kim M (2021). Switchable photonic bio-adhesive materials. Adv. Mater..

[50] Zheng ZG (2017). Light-patterned crystallographic direction of a self-organized 3D soft photonic crystal. Adv. Mater..

[51] Zhang XF (2020). Electro-and photo-driven orthogonal switching of a helical superstructure enabled by an axially chiral molecular switch. ACS Appl. Mater. Interfaces.

[52] Ma LL (2019). Light-activated liquid crystalline hierarchical architecture toward photonics. Adv. Optical Mater..

[53] Tadepalli S (2017). Bio-optics and bio-inspired optical materials. Chem. Rev..

[54] Guidetti G, Omenetto FG (2020). *N*-dimensional optics with natural materials. MRS Commun..

[55] Xiong R (2020). Biopolymeric photonic structures: Design, fabrication, and emerging applications. Chem. Soc. Rev..

[56] Lu YQ, Li Y (2021). Planar liquid crystal polarization optics for near-eye displays. Light Sci. Appl..

[57] Jiang YF (2018). Image flickering-free polymer stabilized fringe field switching liquid crystal display. Opt. Express.

[58] Borshch V, Shiyanovskii SV, Lavrentovich OD (2013). Nanosecond electro-optic switching of a liquid crystal. Phys. Rev. Lett..

[59] Borshch V (2014). Nanosecond electro-optics of a nematic liquid crystal with negative dielectric anisotropy. Phys. Rev. E.

[60] Li BX (2014). Electro-optic switching of dielectrically negative nematic through nanosecond electric modification of order parameter. Appl. Phys. Lett..

[61] Li X (2018). Fast switchable dual-model grating by using polymer-stabilized sphere phase liquid crystal. Polymers.

[62] Foster D (2019). Two-dimensional skyrmion bags in liquid crystals and ferromagnets. Nat. Phys..

[63] Tai JSB, Ackerman PJ, Smalyukh II (2018). Topological transformations of Hopf solitons in chiral ferromagnets and liquid crystals. Proc. Natl Acad. Sci. USA.

[64] Yin K (2020). Patterning liquid-crystal alignment for ultrathin flat optics. ACS Omega.

[65] Kim DS (2018). Mosaics of topological defects in micropatterned liquid crystal textures. Sci. Adv..

[66] Xia Y (2019). Programming emergent symmetries with saddle-splay elasticity. Nat. Commun..

[67] Wei BY (2014). Generating switchable and reconfigurable optical vortices *via* photopatterning of liquid crystals. Adv. Mater..

[68] Akiyama H (2002). Synthesis and properties of azo dye aligning layers for liquid crystal cells. Liq. Cryst..

[69] Wu H (2012). Arbitrary photo-patterning in liquid crystal alignments using DMD based lithography system. Opt. Express.

[70] Duan W (2022). Patterned optical anisotropic film for generation of non-diffracting vortex beams. Appl. Phys. Lett..

[71] Hu W (2012). Polarization independent liquid crystal gratings based on orthogonal photoalignments. Appl. Phys. Lett..

[72] Guo YB (2016). High-resolution and high-throughput plasmonic photopatterning of complex molecular orientations in liquid crystals. Adv. Mater..

[73] Martinez A, Mireles HC, Smalyukh II (2011). Large-area optoelastic manipulation of colloidal particles in liquid crystals using photoresponsive molecular surface monolayers. Proc. Natl Acad. Sci. USA.

[74] Shen Y, Dierking I (2020). Dynamic dissipative solitons in nematics with positive anisotropies. Soft Matter.

[75] Li BX (2020). Soliton-induced liquid crystal enabled electrophoresis. Phys. Rev. Res..

[76] Shen Y, Dierking I (2020). Dynamics of electrically driven solitons in nematic and cholesteric liquid crystals. Commun. Phys..

[77] Satoshi A, Fumito A (2020). Kinetics of motile solitons in nematic liquid crystals. Nat. Commun..

[78] Li BX (2018). Electrically driven three-dimensional solitary waves as director bullets in nematic liquid crystals. Nat. Commun..

[79] Li BX (2019). Three-dimensional solitary waves with electrically tunable direction of propagation in nematics. Nat. Commun..

[80] Kim YH (2011). Smectic liquid crystal defects for self-assembling of building blocks and their lithographic applications. Adv. Funct. Mater..

[81] Kim DS (2015). Fabrication of periodic nanoparticle clusters using a soft lithographic template. J. Mater. Chem. C..

[82] Kléman M (1989). Defects in liquid crystals. Rep. Prog. Phys..

[83] Williams CE, Kléman M (1975). Dislocations, grain boundaries and focal conics in smectics A. Le. J. de. Phys. Colloq..

[84] Honglawan A (2013). Topographically induced hierarchical assembly and geometrical transformation of focal conic domain arrays in smectic liquid crystals. Proc. Natl Acad. Sci. U. State. Am..

[85] Honglawan A (2011). Pillar-assisted epitaxial assembly of toric focal conic domains of smectic-a liquid crystals. Adv. Mater..

[86] Lavrentovich OD, Kléman M, Pergamenshchik VM (1994). Nucleation of focal conic domains in smectic A liquid crystals. J. de. Phys. II.

[87] Kléman M, Lavrentovich OD (2009). Liquids with conics. Liq. Cryst..

[88] Zappone B (2012). Periodic lattices of frustrated focal conic defect domains in smectic liquid crystal films. Soft Matter.

[89] Guo W, Bahr C (2009). Influence of anchoring strength on focal conic domains in smectic films. Phys. Rev. E.

[90] Kim YH (2010). Fabrication of two-dimensional dimple and conical microlens arrays from a highly periodic toroidal-shaped liquid crystal defect array. J. Mater. Chem..

[91] Beller DA (2013). Focal conic flower textures at curved interfaces. Phys. Rev. X.

[92] Yoo HW (2013). Plasmonic three-dimensional dimpled array from highly ordered self-assembled liquid crystal defects. J. Mater. Chem. C..

[93] Yoon DK (2007). Internal structure visualization and lithographic use of periodic toroidal holes in liquid crystals. Nat. Mater..

[94] Gharbi MA (2015). Smectic gardening on curved landscapes. Langmuir.

[95] Ma LL (2017). Smectic layer origami via preprogrammed photoalignment. Adv. Mater..

[96] Kim YH (2010). Optically selective microlens photomasks using self-assembled smectic liquid crystal defect arrays. Adv. Mater..

[97] Serra F (2015). Curvature-driven, one-step assembly of reconfigurable smectic liquid crystal “compound eye” lenses. Adv. Optical Mater..

[98] Ma LL (2019). Self-assembled asymmetric microlenses for four-dimensional visual imaging. ACS Nano.

[99] Son B (2014). Optical vortex arrays from smectic liquid crystals. Opt. Express.

[100] Preusse RS (2020). Hierarchical assembly of smectic liquid crystal defects at undulated interfaces. Soft Matter.

[101] Wu SB (2020). Smectic defect engineering enabled by programmable photoalignment. Adv. Optical Mater..

[102] Gim MJ, Beller DA, Yoon DK (2017). Morphogenesis of liquid crystal topological defects during the nematic-smectic A phase transition. Nat. Commun..

[103] Zappone B (2020). Analogy between periodic patterns in thin smectic liquid crystal films and the intermediate state of superconductors. Proc. Natl Acad. Sci. U. State. Am..

[104] De Gennes PG (1972). An analogy between superconductors and smectics A. Solid State Commun..

[105] Kim DS (2016). Controlling gaussian and mean curvatures at microscale by sublimation and condensation of smectic liquid crystals. Nat. Commun..

[106] Vitral E, Leo PH, Viñals J (2021). Phase-field model for a weakly compressible soft layered material: Morphological transitions on smectic-isotropic interfaces. Soft Matter.

[107] Boniello G (2021). Making smectic defect patterns electrically reversible and dynamically tunable using in situ polymer-templated nematic liquid crystals. Macromol. Rapid Commun..

[108] Lindahl T (1993). Instability and decay of the primary structure of DNA. Nature.

[109] Boyarsky A, Fröhlich J, Ruchayskiy O (2012). Self-consistent evolution of magnetic fields and chiral asymmetry in the early universe. Phys. Rev. Lett..

[110] Wang Y (2013). Emerging chirality in nanoscience. Chem. Soc. Rev..

[111] Zhang SC (2017). Arrays of horizontal carbon nanotubes of controlled chirality grown using designed catalysts. Nature.

[112] Zhang L (2016). Chiral nanoarchitectonics: towards the design, self-assembly, and function of nanoscale chiral twists and helices. Adv. Mater..

[113] De Vries H (1951). Rotatory power and other optical properties of certain liquid crystals. Acta Crystallogr..

[114] Du F (2004). Polymer-stabilized cholesteric liquid crystal for polarization-independent variable optical attenuator. Jpn. J. Appl. Phys..

[115] Li JT (2019). 1,2-dithienyldicyanoethene-based, visible-light-driven, chiral fluorescent molecular switch: Rewritable multimodal photonic devices. Angew. Chem. Int. Ed..

[116] Xiang J (2016). Electrically tunable laser based on oblique heliconical cholesteric liquid crystal. Proc. Natl Acad. Sci. U. State. Am..

[117] Rupnik PM (2017). Field-controlled structures in ferromagnetic cholesteric liquid crystals. Sci. Adv..

[118] Kizhakidathazhath R (2020). Facile anisotropic deswelling method for realizing large-area cholesteric liquid crystal elastomers with uniform structural color and broad-range mechanochromic response. Adv. Funct. Mater..

[119] Stumpel JE (2015). Stimuli-responsive materials based on interpenetrating polymer liquid crystal hydrogels. Adv. Funct. Mater..

[120] Ma LL (2022). Submicrosecond electro-optical switching of one-dimensional soft photonic crystals. Photonics Res..

[121] Ma LL (2015). Rationally designed dynamic superstructures enabled by photoaligning cholesteric liquid crystals. Adv. Optical Mater..

[122] Zheng ZG (2016). Three-dimensional control of the helical axis of a chiral nematic liquid crystal by light. Nature.

[123] Zheng ZG (2017). Controllable dynamic zigzag pattern formation in a soft helical superstructure. Adv. Mater..

[124] Chen LJ (2014). Photoresponsive monodisperse cholesteric liquid crystalline microshells for tunable omnidirectional lasing enabled by a visible light-driven chiral molecular switch. Adv. Optical Mater..

[125] Jiang SA (2021). Control of large-area orderliness of a 2D supramolecular chiral microstructure by a 1D interference field. ACS Appl. Mater. Interfaces.

[126] Helfrich W (1971). Electrohydrodynamic and dielectric instabilities of cholesteric liquid crystals. J. Chem. Phys..

[127] Xiang J (2014). Electrooptic response of chiral nematic liquid crystals with oblique helicoidal director. Phys. Rev. Lett..

[128] Salili SM (2016). Magnetically tunable selective reflection of light by heliconical cholesterics. Phys. Rev. E.

[129] Nava G (2019). Pitch tuning induced by optical torque in heliconical cholesteric liquid crystals. Phys. Rev. Res..

[130] Yuan CL (2019). Stimulated transformation of soft helix among helicoidal, heliconical, and their inverse helices. Sci. Adv..

[131] Kikuchi H (2002). Polymer-stabilized liquid crystal blue phases. Nat. Mater..

[132] Yang YZ (2021). 3D chiral photonic nanostructures based on blue-phase liquid crystals. Small Sci..

[133] Gandhi SS, Chien LC (2017). Unraveling the mystery of the blue fog: structure, properties, and applications of amorphous blue phase III. Adv. Mater..

[134] Castles F (2014). Stretchable liquid-crystal blue-phase gels. Nat. Mater..

[135] Kikuchi, H. Liquid crystalline blue phases. in Liquid Crystalline Functional Assemblies and Their Supramolecular Structures (ed Kato, T.). (Berlin: Springer, 2007), 99-117.

[136] Yan J (2013). A full-color reflective display using polymer-stabilized blue phase liquid crystal. Appl. Phys. Lett..

[137] Choi H (2012). Polymer-stabilized supercooled blue phase. Appl. Phys. Lett..

[138] Lin JD (2018). Microstructure-stabilized blue phase liquid crystals. ACS Omega.

[139] Coles HJ, Pivnenko MN (2005). Liquid crystal ‘blue phases’ with a wide temperature range. Nature.

[140] Wang L (2012). Wide blue phase range and electro-optical performances of liquid crystalline composites doped with thiophene-based mesogens. J. Mater. Chem..

[141] Zhu G (2011). Liquid crystal blue phase induced by bent-shaped molecules with allylic end groups. Optical Mater. Express.

[142] Wang J (2016). Stabilization and electro-optical switching of liquid crystal blue phases using unpolymerized and polymerized polyoxometalate-based nanoparticles. Mol. Cryst. Liq. Cryst..

[143] Xiang J, Lavrentovich OD (2013). Blue-phase-polymer-templated nematic with sub-millisecond broad-temperature range electro-optic switching. Appl. Phys. Lett..

[144] Chen CW (2017). Large three-dimensional photonic crystals based on monocrystalline liquid crystal blue phases. Nat. Commun..

[145] Bukusoglu E (2017). Strain-induced alignment and phase behavior of blue phase liquid crystals confined to thin films. Soft Matter.

[146] Zhou K (2018). Light-driven reversible transformation between self-organized simple cubic lattice and helical superstructure enabled by a molecular switch functionalized nanocage. Adv. Mater..

[147] Schlafmann KR, White TJ (2021). Retention and deformation of the blue phases in liquid crystalline elastomers. Nat. Commun..

[148] Wang M (2017). Asymmetric tunable photonic bandgaps in self-organized 3D nanostructure of polymer-stabilized blue phase I modulated by voltage polarity. Adv. Funct. Mater..

[149] Chen Y, Wu ST (2013). Electric field-induced monodomain blue phase liquid crystals. Appl. Phys. Lett..

[150] Hu W (2020). Humidity-responsive blue phase liquid-crystalline film with reconfigurable and tailored visual signals. Adv. Funct. Mater..

[151] Yang YZ (2021). Bioinspired color-changing photonic polymer coatings based on three-dimensional blue phase liquid crystal networks. ACS Appl. Mater. Interfaces.

[152] Hur ST (2013). Liquid-crystalline blue phase laser with widely tunable wavelength. Adv. Mater..

[153] Almeida APC (2018). Cellulose-based biomimetics and their applications. Adv. Mater..

[154] Xu CL, Huang CX, Huang HH (2021). Recent advances in structural color display of cellulose nanocrystal materials. Appl. Mater. Today.

[155] Peng ZW (2020). Applications of cellulose nanomaterials in stimuli-responsive optics. J. Agric. Food Chem..

[156] Smalyukh II (2021). Thermal management by engineering the alignment of nanocellulose. Adv. Mater..

[157] Frka-Petesic B, Vignolini S (2019). So much more than paper. Nat. Photonics.

[158] Gilbert RD, Patton PA (1983). Liquid crystal formation in cellulose and cellulose derivatives. Prog. Polym. Sci..

[159] Chan CLC (2019). Visual appearance of chiral nematic cellulose-based photonic films: angular and polarization independent color response with a twist. Adv. Mater..

[160] Lagerwall JPF (2014). Cellulose nanocrystal-based materials: from liquid crystal self-assembly and glass formation to multifunctional thin films. NPG Asia Mater..

[161] Joubert F (2014). The preparation of graft copolymers of cellulose and cellulose derivatives using atrp under homogeneous reaction conditions. Chem. Soc. Rev..

[162] Parker RM (2018). The self-assembly of cellulose nanocrystals: hierarchical design of visual appearance. Adv. Mater..

[163] Tran A, Boott CE, MacLachlan MJ (2020). Understanding the self-assembly of cellulose nanocrystals-Toward chiral photonic materials. Adv. Mater..

[164] Revol JF (1992). Helicoidal self-ordering of cellulose microfibrils in aqueous suspension. Int. J. Biol. Macromolecules.

[165] Usov I (2015). Understanding nanocellulose chirality and structure–properties relationship at the single fibril level. Nat. Commun..

[166] Orts WJ (1998). Enhanced ordering of liquid crystalline suspensions of cellulose microfibrils: A small angle neutron scattering study. Macromolecules.

[167] Conley K (2016). Origin of the twist of cellulosic materials. Carbohydr. Polym..

[168] Revol JF, Godbout L, Gray DG (1998). Solid self-assembled films of cellulose with chiral nematic order and optically variable properties. J. Pulp Pap. Sci..

[169] Werbowyj RS, Gray DG (1984). Optical properties of hydroxypropyl cellulose liquid crystals. I. Cholesteric pitch and polymer concentration. Macromolecules.

[170] Espinha A (2018). Hydroxypropyl cellulose photonic architectures by soft nanoimprinting lithography. Nat. Photonics.

[171] Chu G (2019). Printing flowers? Custom-tailored photonic cellulose films with engineered surface topography. Matter.

[172] Cao YY (2020). Tunable diffraction gratings from biosourced lyotropic liquid crystals. Adv. Mater..

[173] Zhang ZH (2022). Cholesteric cellulose liquid crystals with multifunctional structural colors. Adv. Funct. Mater..

[174] Zhao GM (2020). Dual response of photonic films with chiral nematic cellulose nanocrystals: humidity and formaldehyde. ACS Appl. Mater. Interfaces.

[175] Yao K (2017). Flexible and responsive chiral nematic cellulose nanocrystal/poly(ethylene glycol) composite films with uniform and tunable structural color. Adv. Mater..

[176] Meng YH (2020). Fabrication of environmental humidity-responsive iridescent films with cellulose nanocrystal/polyols. Carbohydr. Polym..

[177] Lu T (2017). Cellulose nanocrystals/polyacrylamide composites of high sensitivity and cycling performance to gauge humidity. ACS Appl. Mater. Interfaces.

[178] Qu D (2019). Chiral photonic cellulose films enabling mechano/chemo responsive selective reflection of circularly polarized light. Adv. Optical Mater..

[179] Zhang Y (2020). Responsive and patterned cellulose nanocrystal films modified by N-methylmorpholine-*N*-oxide. Carbohydr. Polym..

[180] Yu HL (2020). Stimuli-responsive circularly polarized luminescent films with tunable emission. J. Mater. Chem. C..

[181] Dai SD (2017). Cholesteric film of Cu(II)-doped cellulose nanocrystals for colorimetric sensing of ammonia gas. Carbohydr. Polym..

[182] Jiang YC (2022). Highly strong luminescent chiral nematic cellulose nanocrystal/PEI composites for anticounterfeiting. Chem. Eng. J..

[183] Kose O (2019). Unwinding a spiral of cellulose nanocrystals for stimuli-responsive stretchable optics. Nat. Commun..

[184] Xu MC (2018). Multifunctional chiral nematic cellulose nanocrystals/glycerol structural colored nanocomposites for intelligent responsive films, photonic inks and iridescent coatings. J. Mater. Chem. C..

[185] Guidetti G (2016). Flexible photonic cellulose nanocrystal films. Adv. Mater..

[186] Boott CE (2020). Cellulose nanocrystal elastomers with reversible visible color. Angew. Chem. Int. Ed..

[187] Qu D (2019). Modulating the structural orientation of nanocellulose composites through mechano-stimuli. ACS Appl. Mater. Interfaces.

[188] Nan FC (2017). Enhanced toughness and thermal stability of cellulose nanocrystal iridescent films by alkali treatment. ACS Sustain. Chem. Eng..

[189] Pal RK (2015). Biopatterning of silk proteins for soft micro-optics. ACS Appl. Mater. Interfaces.

[190] Liang HL (2018). Roll-to-roll fabrication of touch-responsive cellulose photonic laminates. Nat. Commun..

[191] Wang W (2008). Thin films of poly(*N*-isopropylacrylamide) end-capped with *n*-butyltrithiocarbonate. Macromolecules.

[192] Risteen B (2018). Thermally switchable liquid crystals based on cellulose nanocrystals with patchy polymer grafts. Small.

[193] Dumanli AG, Savin T (2016). Recent advances in the biomimicry of structural colours. Chem. Soc. Rev..

[194] Shang LR (2019). Bio-inspired intelligent structural color materials. Mater. Horiz..

[195] Wang H (2018). Photochemically and thermally driven full-color reflection in a self-organized helical superstructure enabled by a halogen-bonded chiral molecular switch. Angew. Chem. Int. Ed..

[196] Qin L (2018). Piecewise phototuning of self-organized helical superstructures. Adv. Mater..

[197] Qin L (2021). Geminate labels programmed by two-tone microdroplets combining structural and fluorescent color. Nat. Commun..

[198] Thapa K (2021). Combined electric and photocontrol of selective light reflection at an oblique helicoidal cholesteric liquid crystal doped with azoxybenzene derivative. Phys. Rev. E.

[199] Kim SU (2022). Broadband and pixelated camouflage in inflating chiral nematic liquid crystalline elastomers. Nat. Mater..

[200] Kragt AJJ (2019). 3D helix engineering in chiral photonic materials. Adv. Mater..

[201] Zhang P (2020). A patterned mechanochromic photonic polymer for reversible image reveal. Adv. Mater. Interfaces.

[202] Schmidtke J, Kniesel S, Finkelmann H (2005). Probing the photonic properties of a cholesteric elastomer under biaxial stress. Macromolecules.

[203] Chen RL (2021). Re-printable chiral photonic paper with invisible patterns and tunable wettability. Adv. Funct. Mater..

[204] Yi H (2019). Ultra-adaptable and wearable photonic skin based on a shape-memory, responsive cellulose derivative. Adv. Funct. Mater..

[205] Deng B (2019). Scalable and ultrafast epitaxial growth of single-crystal graphene wafers for electrically tunable liquid-crystal microlens arrays. Sci. Bull..

[206] Wang YJ (2021). High-efficiency broadband achromatic metalens for near-IR biological imaging window. Nat. Commun..

[207] Chu F (2021). Four-mode 2D/3D switchable display with a 1D/2D convertible liquid crystal lens array. Opt. Express.

[208] Pancharatnam S (1956). Generalized theory of interference and its applications. Proc. Indian Acad. Sci.-Sect. A.

[209] Berry MV (1984). Quantal phase factors accompanying adiabatic changes. Proc. R. Soc. Lond. A. Math. Phys. Sci..

[210] Minovich AE (2015). Functional and nonlinear optical metasurfaces. Laser Photonics Rev..

[211] Shen ZX (2019). Liquid crystal tunable terahertz lens with spin-selected focusing property. Opt. Express.

[212] Jiang M (2019). Low *f*-number diffraction-limited Pancharatnam–Berry microlenses enabled by plasmonic photopatterning of liquid crystal polymers. Adv. Mater..

[213] Dai HT (2015). Optically isotropic, electrically tunable liquid crystal droplet arrays formed by photopolymerization-induced phase separation. Opt. Lett..

[214] Xu Q, Sun T, Wang C (2021). Coded liquid crystal metasurface for achromatic imaging in the broadband wavelength range. ACS Appl. Nano Mater..

[215] Li Y, Zhan T, Wu ST (2020). Flat cholesteric liquid crystal polymeric lens with low *f*-number. Opt. Express.

[216] Zhan T (2020). Practical chromatic aberration correction in virtual reality displays enabled by cost-effective ultra-broadband liquid crystal polymer lenses. Adv. Optical Mater..

[217] Shen ZX (2020). Liquid crystal integrated metalens with tunable chromatic aberration. Adv. Photonics.

[218] Zou JY, Li LS, Wu ST (2022). Gaze-matched pupil steering Maxwellian-view augmented reality display with large angle diffractive liquid crystal lenses. Adv. Photonics Res..

[219] Yin K, He ZQ, Wu ST (2020). Reflective polarization volume lens with small *f*-number and large diffraction angle. Adv. Optical Mater..

[220] Perera K (2021). Converging microlens array using nematic liquid crystals doped with chiral nanoparticles. ACS Appl. Mater. Interfaces.

[221] Tong L (2020). Stable mid-infrared polarization imaging based on quasi-2D tellurium at room temperature. Nat. Commun..

[222] Rubin NA (2019). Matrix fourier optics enables a compact full-stokes polarization camera. Science.

[223] Liu F (2018). Deeply seeing through highly turbid water by active polarization imaging. Opt. Lett..

[224] Rubin NA (2022). Imaging polarimetry through metasurface polarization gratings. Opt. Express.

[225] Savo S, Shrekenhamer D, Padilla WJ (2014). Liquid crystal metamaterial absorber spatial light modulator for THz applications. Adv. Optical Mater..

[226] Chen P (2015). Arbitrary and reconfigurable optical vortex generation: a high-efficiency technique using director-varying liquid crystal fork gratings. Photonics Res..

[227] Zhang ZC, You Z, Chu DP (2014). Fundamentals of phase-only liquid crystal on silicon (LCOS) devices. Light Sci. Appl..

[228] Kobashi J, Yoshida H, Ozaki M (2016). Planar optics with patterned chiral liquid crystals. Nat. Photonics.

[229] Chiang WF (2020). Continuously tunable intensity modulators with large switching contrasts using liquid crystal elastomer films that are deposited with terahertz metamaterials. Opt. Express.

[230] Wang L (2015). Broadband tunable liquid crystal terahertz waveplates driven with porous graphene electrodes. Light Sci. Appl..

[231] Shen YJ (2019). Optical vortices 30 years on: OAM manipulation from topological charge to multiple singularities. Light Sci. Appl..

[232] Li ZX (2021). Liquid crystal devices for vector vortex beams manipulation and quantum information applications. Chin. Opt. Lett..

[233] Padgett M, Bowman R (2011). Tweezers with a twist. Nat. Photonics.

[234] Milione G (2015). Using the nonseparability of vector beams to encode information for optical communication. Opt. Lett..

[235] García-Escartín JC, Chamorro-Posada P (2011). Universal quantum computation with the orbital angular momentum of a single photon. J. Opt..

[236] Foo G, Palacios DM, Swartzlander GA (2005). Optical vortex coronagraph. Opt. Lett..

[237] Yang HW (2021). Steering nonlinear twisted valley photons of monolayer WS_2_ by vector beams. Nano Lett..

[238] Wang XW (2018). Recent advances on optical vortex generation. Nanophotonics.

[239] Ni JC (2017). Three-dimensional chiral microstructures fabricated by structured optical vortices in isotropic material. Light Sci. Appl..

[240] Kobashi J, Yoshida H, Ozaki M (2016). Polychromatic optical vortex generation from patterned cholesteric liquid crystals. Phys. Rev. Lett..

[241] Chen P (2018). Digitalizing self-assembled chiral superstructures for optical vortex processing. Adv. Mater..

[242] Brasselet, E. Singular optics of liquid crystal defects. in Liquid Crystals: New Perspectives (eds Pieranski P. and Godinho M. H.). (Hoboken: John Wiley & Sons, 2021), 1-79.

[243] Ackerman PJ (2012). Laser-directed hierarchical assembly of liquid crystal defects and control of optical phase singularities. Sci. Rep..

[244] Voloschenko D, Lavrentovich OD (2000). Optical vortices generated by dislocations in a cholesteric liquid crystal. Opt. Lett..

[245] Nassiri MG, Brasselet E (2018). Multispectral management of the photon orbital angular momentum. Phys. Rev. Lett..

[246] Brasselet E (2009). Optical vortices from liquid crystal droplets. Phys. Rev. Lett..

[247] Papič M (2021). Topological liquid crystal superstructures as structured light lasers. Proc. Natl Acad. Sci. USA.

[248] Chen J (2021). Experimental demonstration of cylindrical vector spatiotemporal optical vortex. Nanophotonics.

[249] Rechcińska K (2019). Engineering spin-orbit synthetic hamiltonians in liquid-crystal optical cavities. Science.

[250] Zuo B (2019). Visible and infrared three-wavelength modulated multi-directional actuators. Nat. Commun..

[251] Huang YL (2021). Bioinspired synergistic photochromic luminescence and programmable liquid crystal actuators. Angew. Chem. Int. Ed..

[252] Li Y, Liu YJ, Luo D (2021). Polarization dependent light-driven liquid crystal elastomer actuators based on photothermal effect. Adv. Optical Mater..

[253] Gelebart AH (2017). Making waves in a photoactive polymer film. Nature.

[254] Ware TH (2015). Voxelated liquid crystal elastomers. Science.

[255] Sawa Y (2013). Shape and chirality transitions in off-axis twist nematic elastomer ribbons. Phys. Rev. E.

[256] Feng CR, Rajapaksha CPH, Jákli A (2021). Ionic elastomers for electric actuators and sensors. Engineering.

[257] Feng W, Broer DJ, Liu DQ (2018). Oscillating chiral-nematic fingerprints wipe away dust. Adv. Mater..

[258] Hu WQ (2018). Small-scale soft-bodied robot with multimodal locomotion. Nature.

[259] Feng W, Liu DQ, Broer DJ (2020). Functional liquid crystal polymer surfaces with switchable topographies. Small Struct..

[260] Zeng H (2017). Self-regulating iris based on light-actuated liquid crystal elastomer. Adv. Mater..

[261] Cheng M (2022). Light-fueled polymer film capable of directional crawling, friction-controlled climbing, and self-sustained motion on a human hair. Adv. Sci..

[262] Babakhanova G (2018). Liquid crystal elastomer coatings with programmed response of surface profile. Nat. Commun..

[263] Babakhanova G (2019). Controlled placement of microparticles at the water-liquid crystal elastomer interface. ACS Appl. Mater. Interfaces.

[264] Eelkema R (2006). Molecular machines: Nanomotor rotates microscale objects. Nature.

[265] Ma LL (2017). Light-driven rotation and pitch tuning of self-organized cholesteric gratings formed in a semi-free film. Polymers.

[266] Yuan Y (2019). Elastic colloidal monopoles and reconfigurable self-assembly in liquid crystals. Nature.

[267] Yuan Y (2018). Self-assembled nematic colloidal motors powered by light. Nat. Commun..

[268] Zhou H (2018). Bio-inspired photonic materials: Prototypes and structural effect designs for applications in solar energy manipulation. Adv. Funct. Mater..

[269] Khan MK, Hamad WY, MacLachlan MJ (2014). Tunable mesoporous bilayer photonic resins with chiral nematic structures and actuator properties. Adv. Mater..

[270] Gladman AS (2016). Biomimetic 4D printing. Nat. Mater..

[271] Bettotti P (2016). Dynamics of hydration of nanocellulose films. Adv. Mater. Interfaces.

[272] Wang M (2015). Sensitive humidity-driven reversible and bidirectional bending of nanocellulose thin films as bio-inspired actuation. Adv. Mater. Interfaces.

[273] Mredha MTI (2019). Anisotropic tough multilayer hydrogels with programmable orientation. Mater. Horiz..

[274] Cao J (2020). Ultrarobust Ti_3_C_2_T_*x*_ MXene-based soft actuators *via* bamboo-inspired mesoscale assembly of hybrid nanostructures. ACS Nano.

[275] Wu TH (2016). A bio-inspired cellulose nanocrystal-based nanocomposite photonic film with hyper-reflection and humidity-responsive actuator properties. J. Mater. Chem. C..

[276] Wang SC (2021). Scalable thermochromic smart windows with passive radiative cooling regulation. Science.

[277] Khandelwal H, Schenning APHJ, Debije MG (2017). Infrared regulating smart window based on organic materials. Adv. Energy Mater..

[278] Yang JJ (2021). Beyond the visible: bioinspired infrared adaptive materials. Adv. Mater..

[279] Wang JQ (2021). A fully self-powered, ultra-stable cholesteric smart window triggered by instantaneous mechanical stimuli. Nano Energy.

[280] Xia Y (2019). High-efficiency and reliable smart photovoltaic windows enabled by multiresponsive liquid crystal composite films and semi-transparent perovskite solar cells. Adv. Energy Mater..

[281] Kim M (2015). Fabrication of microcapsules for dye-doped polymer-dispersed liquid crystal-based smart windows. ACS Appl. Mater. Interfaces.

[282] Murray J, Ma DK, Munday JN (2017). Electrically controllable light trapping for self-powered switchable solar windows. ACS Photonics.

[283] Yoon WJ (2020). A single-step dual stabilization of smart window by the formation of liquid crystal physical gels and the construction of liquid crystal chambers. Adv. Funct. Mater..

[284] Gutierrez-Cuevas KG (2016). Frequency-driven self-organized helical superstructures loaded with mesogen-grafted silica nanoparticles. Angew. Chem. Int. Ed..

[285] Wang L (2017). Stimuli-directed self-organized chiral superstructures for adaptive windows enabled by mesogen-functionalized graphene. Mater. Today.

[286] Liang X (2017). A roll-to-roll process for multi-responsive soft-matter composite films containing Cs_*x*_WO_3_ nanorods for energy-efficient smart window applications. Nanoscale Horiz..

[287] De La Cruz JA (2018). Cellulose-based reflective liquid crystal films as optical filters and solar gain regulators. ACS Photonics.

[288] Satapathy P (2020). Switchable smart windows using a biopolymer network of cellulose nanocrystals imposed on a nematic liquid crystal. Appl. Phys. Lett..

[289] Chen X (2020). First-principles experimental demonstration of ferroelectricity in a thermotropic nematic liquid crystal: polar domains and striking electro-optics. Proc. Natl Acad. Sci. USA.

[290] Wang Y (2021). Light-activated shape morphing and light-tracking materials using biopolymer-based programmable photonic nanostructures. Nat. Commun..

[291] Wang Y (2017). Modulation of multiscale 3D lattices through conformational control: Painting silk inverse opals with water and light. Adv. Mater..

[292] Wang Y (2019). Controlling silk fibroin conformation for dynamic, responsive, multifunctional, micropatterned surfaces. Proc. Natl Acad. Sci. USA.

[293] Wang Y (2019). Biomaterial-based “structured opals” with programmable combination of diffractive optical elements and photonic bandgap effects. Adv. Mater..

[294] Wang YS, Li M, Wang Y (2020). Silk: a versatile biomaterial for advanced optics and photonics. Chin. Opt. Lett..

[295] Li WY (2019). Inkjet printing of patterned, multispectral, and biocompatible photonic crystals. Adv. Mater..

[296] Jau HC (2015). Light-driven wide-range nonmechanical beam steering and spectrum scanning based on a self-organized liquid crystal grating enabled by a chiral molecular switch. Adv. Optical Mater..

[297] Bisoyi HK, Bunning TJ, Li Q (2018). Stimuli-driven control of the helical axis of self-organized soft helical superstructures. Adv. Mater..

[298] Lee H (2011). 11.1: *Invited Paper*: The world's first blue phase liquid crystal display. SID Symp . Dig. Tech. Pap..

[299] Matsui T (2022). Visualizing invisible phase transitions in blue phase liquid crystals using early warning indicators. Small.

